# Postsynaptic cAMP signalling regulates the antagonistic balance of *Drosophila* glutamate receptor subtypes

**DOI:** 10.1242/dev.191874

**Published:** 2020-12-16

**Authors:** Kai Zhao, Huilin Hong, Lu Zhao, Sheng Huang, Ying Gao, Elsayed Metwally, Yuqiang Jiang, Stephan J. Sigrist, Yong Q. Zhang

**Affiliations:** 1 Key Laboratory for Molecular and Developmental Biology, Institute of Genetics and Developmental Biology, Chinese Academy of Sciences, Beijing 100101, China; 2 Freie Universität Berlin, Institute for Biology/Genetics, Takustrasse 6, 14195 Berlin, Germany; 3 NeuroCure, Charite, Chariteplatz 1, 10117 Berlin, Germany

**Keywords:** Glutamate receptor subtypes, Structured illumination microscopy, Synaptic plasticity, cAMP, *Drosophila*, Neuromuscular junction

## Abstract

The balance among different subtypes of glutamate receptors (GluRs) is crucial for synaptic function and plasticity at excitatory synapses. However, the mechanisms balancing synaptic GluR subtypes remain unclear. Herein, we show that the two subtypes of GluRs (A and B) expressed at *Drosophila* neuromuscular junction synapses mutually antagonize each other in terms of their relative synaptic levels and affect subsynaptic localization of each other, as shown by super-resolution microscopy. Upon temperature shift-induced neuromuscular junction plasticity, GluR subtype A increased but subtype B decreased with a timecourse of hours. Inhibition of the activity of GluR subtype A led to imbalance of GluR subtypes towards more GluRIIA. To gain a better understanding of the signalling pathways underlying the balance of GluR subtypes, we performed an RNA interference screen of candidate genes and found that postsynaptic-specific knockdown of *dunce*, which encodes cAMP phosphodiesterase, increased levels of GluR subtype A but decreased subtype B. Furthermore, bidirectional alterations of postsynaptic cAMP signalling resulted in the same antagonistic regulation of the two GluR subtypes. Our findings thus identify a direct role of postsynaptic cAMP signalling in control of the plasticity-related balance of GluRs.

## Abstract (precis)

**Summary:** The antagonistic balance of GluR subtypes, which is associated with synaptic plasticity, is regulated by cAMP signalling in postsynaptic muscles of *Drosophila* NMJ synapses.

## INTRODUCTION

Ionotropic glutamate receptors (GluRs) are heterotetrameric cation-permeable channels that mediate most excitatory synaptic transmissions in the central nervous system. They can be divided into three large families, namely AMPA receptors (AMPARs), NMDA receptors (NMDARs) and kainate receptors, each of which can be subdivided into several subtypes according to the combination of their subunits (Shepherd and Huganir, 2007; Paoletti et al., 2013). Variation in the composition of synaptic GluR subtypes mediates synaptic plasticity. For example, GluA1 (a subunit of AMPAR) is required for long-term potentiation, whereas GluA2 is involved in long-term depression (Shi et al., 2001; Malinow and Malenka, 2002; Huganir and Nicoll, 2013). Additionally, many neuropsychiatric disorders, such as Alzheimer's disease and Huntington's disease, are linked to dysfunction and imbalance of GluR subtypes (Milnerwood et al., 2010; Malinow, 2012). However, the mechanism controlling the balance of GluR subtypes remains unclear.

The *Drosophila* larval neuromuscular junction (NMJ) is a versatile and effective model system for studying glutamatergic synapses, because the pre- and postsynaptic molecular machinery is similar to that of central excitatory synapses in vertebrates (Harris and Littleton, 2015; Van Vactor and Sigrist, 2017). Fly NMJ GluRs are proposed to be heterotetrameric complexes composed of three essential subunits (GluRIIC, GluRIID and GluRIIE), in addition to either GluRIIA or GluRIIB (also referred to as GluR subtypes A and B hereafter) (Petersen et al., 1997; DiAntonio et al., 1999; Marrus et al., 2004; Featherstone et al., 2005; Qin et al., 2005). Subtype A and B receptors differ in their single-channel properties and synaptic currents (Petersen et al., 1997; DiAntonio et al., 1999). Changes in the balance of GluR subtypes have profound effects on neurotransmission and synaptic plasticity. Notably, elevated synaptic levels of GluRIIA in conjunction with reduced levels of GluRIIB were found to mediate the strengthening of NMJ transmission (Sigrist et al., 2002, 2003).

The cAMP signalling pathway increases the probability of presynaptic vesicle release through enhanced vesicle docking, resulting in short-term memory (Kandel, 2001; Kandel et al., 2014). In addition, cAMP activates the cAMP response element-binding protein (CREB) through protein kinase A (PKA), which activates the expression of downstream genes and mediates long-term memory formation (Kandel, 2001). Studies on *Drosophila* NMJs also showed that cAMP regulates presynaptic vesicle release probability and facilitation (Zhong and Wu, 1991). The first two genes identified in *Drosophila* learning and memory mutants were *dunce* (*dnc*) and *rutabaga* (*rut*). The *dnc* gene encodes a cAMP-specific phosphodiesterase that catalyses the degradation of cAMP, whereas *rut* encodes a type I Ca^2+^/calmodulin-stimulated adenylate cyclase that catalyses the conversion of ATP to cAMP (Davis, 2005). Thus far, cAMP signalling has been shown to function in synaptic plasticity mainly at presynaptic terminals (Kandel, 2001), but little is known about the mechanisms by which cAMP regulates synaptic plasticity on the postsynaptic side.

Although a negative correlation between subtype A and B receptors has been reported at *Drosophila* NMJs (Marrus et al., 2004; Pan and Broadie, 2007; Sulkowski et al., 2014), little is known about the mutual regulation of the two GluR subtypes. To gain a better understanding of the mechanism controlling GluR subtypes at synapses, in this study we characterized the mutual negative regulation of different GluR subtypes and observed that up- or downregulation of one GluR subtype induced the opposite change in the other, while the total synaptic GluR level did not change, indicating an antagonistic balance of GluR subtypes. To gain a better understanding of the mechanism for the antagonistic balance of the two GluR subtypes, we performed an RNA interference (RNAi) screen of candidate genes and found that postsynaptic-specific downregulation of *dnc* induced a synaptic increase in GluR subtype A receptors and a concomitant reduction of subtype B receptors. We also showed that altering cAMP signalling bidirectionally in postsynaptic muscles disrupted the balance of GluR subtypes. In summary, this work reveals an antagonistic balance of different GluR subtypes regulated by cAMP signalling in the postsynaptic compartment at *Drosophila* NMJs, thus offering new insights into the mechanism of synaptic plasticity.

## RESULTS

### GluR subtypes A and B negatively regulate the synaptic abundance of each other

**Fig. 1.**
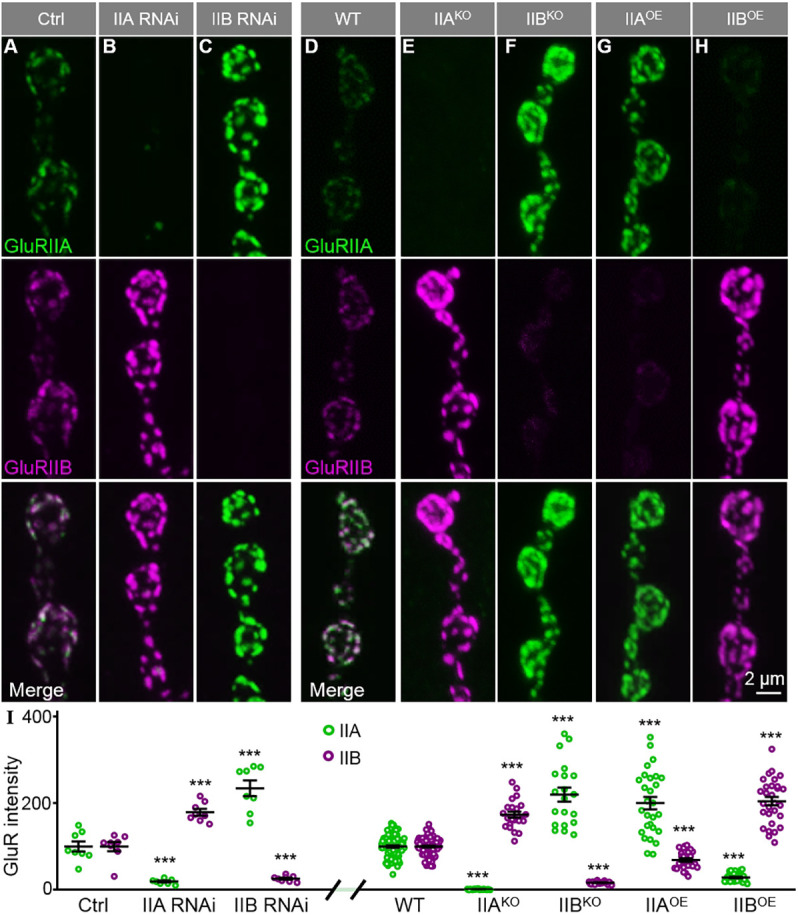
**GluRIIA and GluRIIB negatively regulate each other.** (A–H) Representative confocal images of NMJ4 stained with anti-GluRIIA (green) and anti-GluRIIB (magenta). The full genotypes are as follows: control (Ctrl; *C57-Gal4*, *UAS-Dicer2*/+), IIA RNAi (*C57-Gal4*, *UAS-Dicer2/UAS-GluRIIA-RNAi*), IIB RNAi (*C57-Gal4*, *UAS-Dicer2/UAS-GluRIIB-RNAi*), wild type (*w^1118^*), IIA^KO^ (*GluRIIA^SP16^*), IIB^KO^, IIA^OE^ (*Mhc-GluRIIA*) and IIB^OE^ (*Mhc-GluRIIB*)*.* Scale bar: 2 μm in H. (I) Quantification of the fluorescence intensities of anti-GluRIIA and anti-GluRIIB staining at the NMJs of different genotypes. Data are expressed as percentages of the Ctrl or wild-type fluorescence intensity. *n*=8 NMJs for each genotype in A–C and *n*≥20 for each genotype in D–H. ***P<0.001. Error bars indicate s.e.m.

Although the balance of GluR subtypes is closely related to synaptic plasticity (Huganir and Nicoll, 2013; Paoletti et al., 2013), little is known about the mechanisms underlying the balance of GluR subtypes. To understand how the balance of the GluR subtypes is regulated, we used *Drosophila* NMJ synapses as a model system at which there are only two GluR subtypes, A and B. We assessed the abundance of GluRIIA and GluRIIB over the entire synaptic area, defined by immunostaining with antibodies against horseradish peroxidase (HRP), upon altering the expression of either GluRIIA or GluRIIB. We found that the GluRIIA null mutation or postsynaptic reduction of GluRIIA in muscle cells by RNAi led to GluRIIB accumulation at synapses, and vice versa (Fig. 1A–F). Compared with genetic controls (*C57-Gal4*/+ or wild type), the average synaptic GluRIIA intensity increased by 134.4% for *GluRIIB* RNAi larvae (*P*<0.001, *t*_(14)_=6.17) and by 119.8% for *GluRIIB* null mutants (*P*<0.001, *t*_(39)_=7.10), and the intensity of GluRIIB rose by 79.5% for *GluRIIA* RNAi larvae (*P*<0.001, *t*_(14)_=6.02) and by 73.6% for *GluRIIA* null mutants (*P*<0.001, *t*_(44)_=8.98; Fig. 1I).

A negative correlation between subtype A and B GluRs was also observed when either GluRIIA or GluRIIB was overexpressed (Fig. 1D,G,H). Compared with wild-type controls, synaptic GluRIIA was decreased to 28.8% when GluRIIB was overexpressed (*P*<0.001, *t*_(80)_=14.57), and synaptic GluRIIB was reduced to 69.3% in GluRIIA overexpressing larvae (*P*<0.001, *t*_(79)_=5.79; Fig. 1I). Taken together, these results identify a mutual negative regulation between subtype A and B GluRs at NMJ synapses.

The levels of two GluR subtypes changing in opposite directions suggest that the total GluR level might remain normal at NMJ synapses. To test this possibility, we immunostained GluRIID, one of the essential subunits of the GluR complex. At NMJs of *GluRIIA* or *GluRIIB* null mutants, the intensities of GluRIID were largely unchanged compared with wild-type controls (100±4.48 for wild-type flies; 109.6±6.33 for *GluRIIA* null mutants, *P=*0.408; 85.1±8.61 for *GluRIIB* mull mutants, *P=*0.183; *F*_(2,90)_=0.60; Fig. S1D–F,I). For *GluRIIA* or *GluRIIB* RNAi knockdown larvae, the intensities of GluRIID were also normal (100±10.67 for control flies; 108.6±9.1 for *GluRIIA* RNAi knockdown, *P=*0.808; 104.2±13.38 for *GluRIIB* RNAi knockdown, *P=*0.950; *F*_(2,32)_=0.15; Fig. S1A–C,I). Likewise, the intensity of GluRIID appeared normal upon overexpression (OE) of GluRIIA or GluRIIB (100±6.12 for controls; 104.4±7.01 for *GluRIIA^OE^*, *P=*0.9388; 119±4.33 for *GluRIIB^OE^*, *P=*0.3294; *F*_(2,58)_=0.91; Fig. S1D,G–I). Together, these results show that the synaptic level of GluRIID remains unchanged regardless of altered expression of GluRIIA or GluRIIB, indicating an antagonistic balance between the two GluR subtypes.

### GluR subtypes A and B form concentric ring structures revealed by super-resolution microscopy

**Fig. 2.**
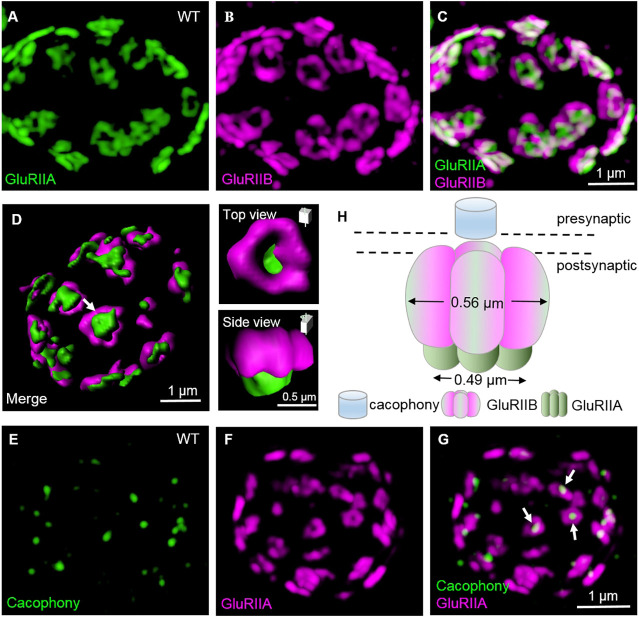
**The subtype B forms a doughnut-shaped ring, with a smaller subtype A ring in the centre.** (A–C) Structured illumination microscopy images of wild-type NMJ4 boutons double stained with anti-GluRIIA (green) and anti-GluRIIB (magenta). Scale bar: 1 μm in C. (D) Three-dimensional surface-rendering images of wild-type synaptic boutons stained with anti-GluRIIA (green) and anti-GluRIIB (magenta). A representative synapse (arrow) is shown in top and side views on the right. (E–G) Structured illumination microscopy images of *elav-GAL4/UAS-cac1-EGFP* NMJ4 boutons double stained with anti-GFP (green) and anti-GluRIIA (magenta). Arrows indicate that Cacophony is juxtaposed to the centre of the GluRIIA ring from the top view. Scale bar: 1 μm in G. (H) Three-dimensional diagram of the localization of Cacophony and GluR subtypes A and B.

Under standard confocal resolution, the GluR subtypes A and B largely overlap as discrete puncta in the NMJ boutons (Marrus et al., 2004; Schmid et al., 2008; Owald et al., 2012). To analyse the subsynaptic localization of subtype A and B receptors, we performed super-resolution imaging using structured illumination microscopy and found that subtype A typically formed a central ring (diameter 0.49±0.01 μm, *n*=322), which was surrounded by a doughnut-shaped outer ring (diameter 0.56±0.01 μm, *n*=324) of subtype B (top view, optical axis perpendicular to the plasma membrane; Fig. 2A–C,H). Furthermore, GluR subtype A and B rings within a synapse did not appear continuous, but instead consisted of several discrete foci (Fig. 2A–C,H). Three-dimensional reconstructions revealed that GluR subtype B rings were localized closer to presynaptic terminals than subtype A rings along the *z*-axis, and the two GluR subtypes together formed a bowl-shaped structure (side view, optical axis parallel to the membrane; Fig. 2D,H). Structured illumination microscopy analysis also showed that Cacophony, a voltage-gated calcium channel located at the centre of the presynaptic active zone (AZ), was juxtaposed at the centre of the GluR subtype A rings (Fig. 2E–G,H). These observations revealed a distinct non-overlapping localization of GluR subtypes A and B at synaptic boutons.

### GluRIIA rings are enlarged when GluRIIA levels are increased at NMJ synapses

**Fig. 3.**
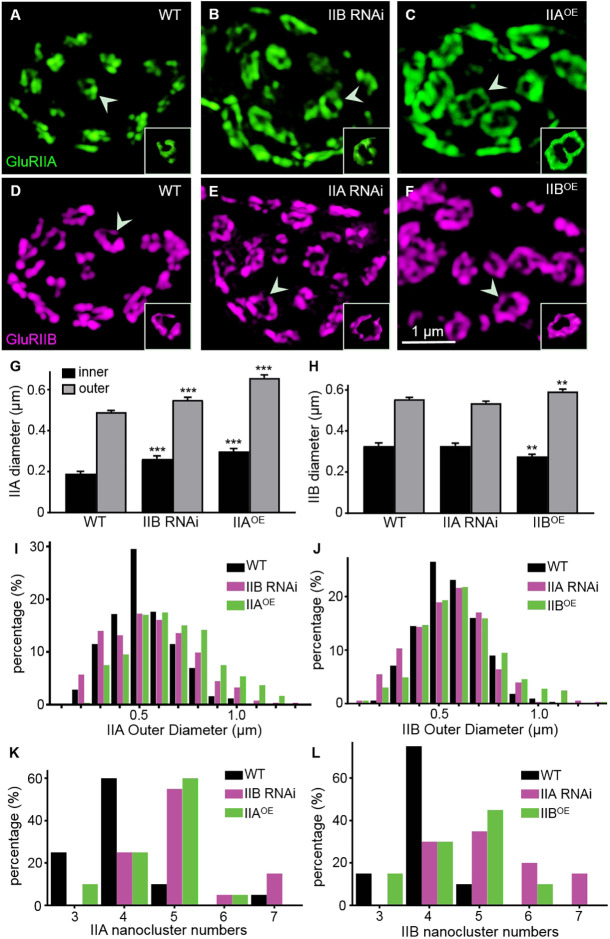
**Enlarged GluRIIA rings when GluRIIA level is increased at NMJ synapses.** (A–F) Representative structured illumination microscopy images of third-instar NMJ4 from different genotypes labelled with anti-GluRIIA (green) and anti-GluRIIB (magenta). The genotypes are as follows: wild type (A,D), IIB RNAi (B), IIA^OE^ (C), IIA RNAi (E) and IIB^OE^ (F)*.* Three-dimensional surface-rendering images of selected GluR rings are shown in the inset (arrowhead points at the selected synapse). Scale bar: 1 μm in F. (G,H) Quantification of inner and outer diameters of the rings of two GluR subtypes in different genotypes. *n*≥44 for inner diameters and *n*≥240 for outer diameters for each genotype. **P<0.01; ***P<0.001. Error bars indicate s.e.m. (I,J) Distributions of outer diameters of the two GluR subtype rings in different genotypes. (K,L) Distribution of the numbers of nanoclusters of each GluR ring in different genotypes.

To study the distinct localization of GluR subtypes in the antagonistic balance of the two GluR subtypes, we next quantified the size of GluRIIA and GluRIIB rings when the expression of either GluRIIA or GluRIIB was altered, using super-resolution structured illumination microscopy imaging. Compared with wild type, the inner and outer diameter of GluR subtype A rings increased significantly upon *GluRIIB* RNAi knockdown or *GluRIIA* overexpression (IIA inner=0.19±0.01 μm for wild type; 0.26±0.01 μm for IIB RNAi knockdown, *P*<0.001; 0.30±0.01 μm for IIA^OE^, *P*<0.001; *F*_(2,141)_=2.79; IIA outer=0.49±0.01 μm for wild type; 0.55±0.01 μm for IIB RNAi knockdown, *P*<0.001; 0.66±0.01 μm for IIA^OE^, *P*<0.001; *F*_(2,890)_=19.39; Fig. 3A–C,G); staining signals for GluRIIB after *GluRIIB* RNAi knockdown or *GluRIIA* overexpression were greatly reduced (Fig. 1) and not analysed here. Distribution analysis of the outer diameter revealed more enlarged subtype A rings in *GluRIIB* RNAi knockdown and *GluRIIA*-overexpressing larvae than in wild-type controls (9.8, 20.2 and 32.9% of IIA rings ≥0.8 μm for wild type, IIB RNAi knockdown and IIA^OE^ flies, respectively; Fig. 3I,J).

Likewise, larvae overexpressing *GluRIIB* displayed slightly but significantly enlarged rings; the outer diameter of subtype B rings was increased compared with wild type (0.56±0.01 μm for wild type, 0.59±0.01 μm for IIB^OE^ flies, *P*=0.007, *t*_(712)_=2.70; Fig. 3D,F,H). However, the inner diameter of subtype B rings in *GluRIIB*-overexpressing larvae was decreased (0.33±0.01 μm for wild type, 0.28±0.01 μm for IIB^OE^ flies, *P=*0.005, *t*_(121)_=2.86; Fig. 3D,F,H), indicating that the width of subtype B rings was increased in *GluRIIB-*overexpressing larvae. Unlike the findings in *GluRIIB-*overexpressing larvae, the inner and outer diameter of subtype B rings in *GluRIIA* RNAi knockdown animals was largely normal, probably because the increase in GluRIIB levels caused by *GluRIIA* knockdown was not large enough to cause a structural change (IIB inner=0.33±0.01 μm for wild type, 0.36±0.02 μm for IIA RNAi knockdown, *P=*0.231, *t*_(73)_=1.21; IIB outer=0.56±0.01 μm for wild type, 0.54±0.01 μm for IIA RNAi knockdown, *P=*0.127, *t*_(762)_=1.53; Fig. 3D,E,H). Together, these results show that an elevated level of GluRIIA caused either by overexpressing GluRIIA or by reducing GluRIIB led to larger subtype A rings, whereas subtype B rings remained normal when GluRIIA was knocked down but were enlarged when GluRIIB was overexpressed.

To describe the GluR rings better, we quantified the nanoclusters of subtype A and B rings for all genotypes (Fig. 3K,L). The results showed that the number of nanoclusters of either subtype A or subtype B receptors was between three and seven. The number of the majority of type A and B receptor nanoclusters was four in wild type (IIA, 60%; IIB, 75%; Fig. 3K,L). However, the number of subtype A receptor nanoclusters was five when GluRIIA was overexpressed or when GluRIIB was knocked down by RNAi (IIA OE, 60%; IIB RNAi, 55%; Fig. 3K). The number of subtype B receptor nanoclusters was also five when GluRIIB was overexpressed or when GluRIIA was knocked down by RNAi (IIB OE, 45%; IIA RNAi, 35%; Fig. 3L). These results indicate that the number of GluR nanoclusters was positively associated with the size of the rings.

### GluRIIA and GluRIIB negatively regulate each other at post-transcriptional level

**Fig. 4.**
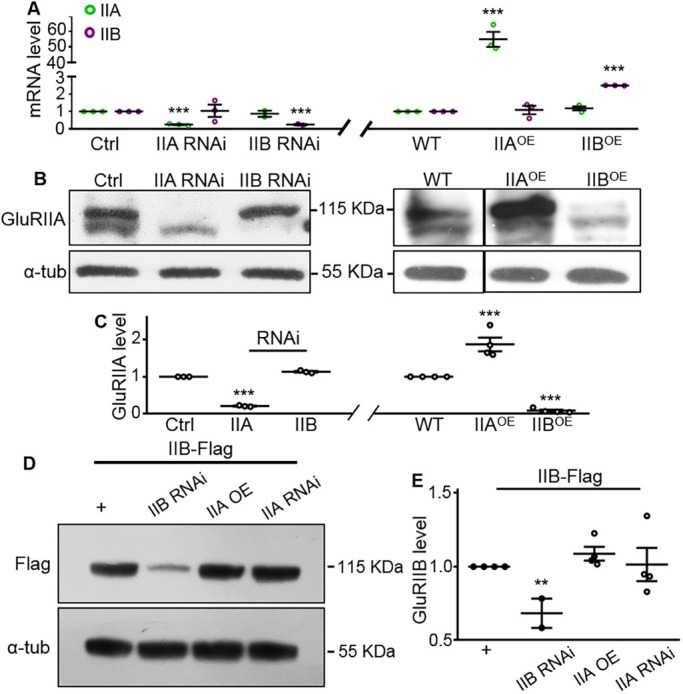
**Negative regulation between GluRIIA and GluRIIB occurs at the post-transcriptional level.** (A) Real-time quantitative PCR analysis of the levels of GluRIIA and GluRIIB mRNA in the larval carcasses of control (Ctrl; *C57-Gal4*, *UAS-Dicer2*/+), IIA RNAi, IIB RNAi, wild type, IIA^OE^ and IIB^OE^ flies. The GluRIIA or GluRIIB mRNA level was normalized to the actin mRNA level. *n*=4. (B,D) Representative western blots of muscle lysates used for quantifying the total amount of GluRIIA and Flag-tagged GluRIIB. The full genotypes in D are as follows: IIB-Flag/+ (*GluRIIB-Flag*/+), IIB-Flag/IIB RNAi (*GluRIIB-Flag*/+*; C57-Gal4*, *UAS-GluRIIB RNAi*/+), IIB-Flag/IIA OE (*Mhc-GluRIIA*, *GluRIIB-Flag*/+), IIB-Flag/IIA RNAi (*GluRIIB-Flag*/+*; C57-Gal4*, *UAS-GluRIIA RNAi*/+). α-tubulin was used as a loading control. (C,E) Quantification of GluRIIA and GluRIIB protein levels normalized to the α-tubulin control in different genotypes. *n*≥3. **P<0.01; ***P<0.001. Error bars indicate s.e.m.

To reveal the level at which the mutual negative regulation of different subtypes of GluRs occurs, we quantified the mRNA and total protein levels of GluRIIA and GluRIIB. Quantitative PCR (qPCR) analysis showed that the mRNA levels of *GluRIIA* or *GluRIIB* did not change regardless of whether the other changed, suggesting that the reciprocal negative regulation of subtype A and B receptors did not occur at the transcriptional level (Fig. 4A).

Compared with the genetic control, total protein levels of GluRIIA were normal when GluRIIB was reduced via RNAi (Fig. 4B,C). By contrast, overexpression of GluRIIB led to a dramatic decrease in total GluRIIA protein to 7.6% of the wild-type control level (Fig. 4B,C). Given that an effective GluRIIB antibody was not available for western blots, we knocked in a Flag tag at the C-terminus of endogenous GluRIIB and examined the GluRIIB protein level with anti-Flag antibody. Co-immunostaining the NMJs of the homozygous *GluRIIB-Flag* lines with antibodies against GluRIIB and anti-GluRIIA or anti-Flag verified the synaptic localization of GluRIIB-Flag (Fig. S2). Initially, we examined the synaptic expression of GluRIIA and GluRIIB-Flag when GluRIIA was reduced via RNAi or increased by overexpression, and we observed a mutual negative regulation between the two GluR subtypes (data not shown). Next, we tested the total protein level of GluRIIB-Flag and found it to be normal no matter how GluRIIA was altered (Fig. 4D,E). Taken together, the qPCR and immunochemical results demonstrate that the reciprocal negative regulation of the two GluR subtypes occurs at NMJ synapses, but not at the total protein level, except for GluRIIB overexpression, which suppressed GluRIIA expression at both NMJ synapses and the total protein level.

### GluRIIA is increased, but GluRIIB is decreased during temperature-dependent plasticity

**Fig. 5.**
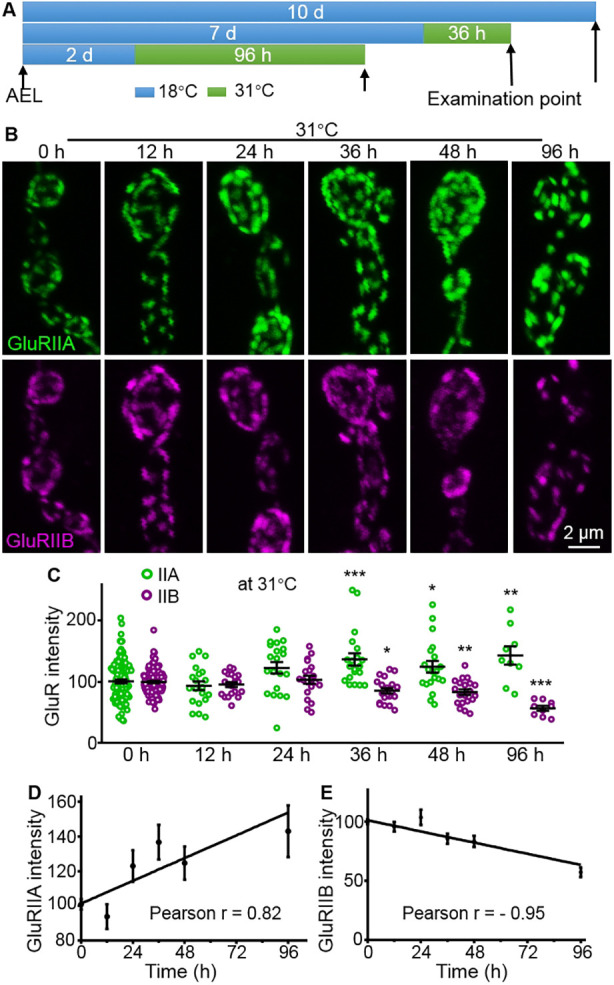
**Negative correlation of GluR subtypes occurs in the process of high temperature-mediated plasticity.** (A) Schematic representation of elevated temperature treatments for different times. wild-type flies were raised at 18°C AEL and transferred to 31°C for different durations until examination at the late third-instar larvae stage (flies develop roughly two times faster at 31°C than at 18°C). d, days. (B) Representative confocal images of NMJ4 co-stained with anti-GluRIIA (green) and anti-GluRIIB (magenta) from late third-instar larvae raised at 31°C for different durations. Scale bar: 2 μm. (C) Statistical analysis of staining intensities of GluRIIA and GluRIIB at the NMJ of late third-instar larvae. *n*≥20 for each time point. *P<0.05; **P<0.01; ***P<0.001. (D,E) Correlation of duration at high temperature and synaptic levels of subtype A (D) or B (E). Error bars indicate s.e.m.

Synaptic plasticity is the ability of synapses to strengthen or weaken in response to increases or decreases of their activity. In *Drosophila*, high temperature-induced plasticity, including overgrowth of NMJs, increased neurotransmitter release and accumulation of GluRIIA receptors, has been identified at NMJs (Sigrist et al., 2003; Zhong and Wu, 2004; Schuster, 2006). However, it is not known whether the balance of GluR subtypes is involved in synaptic plasticity in *Drosophila*. To address this question, we examined synaptic GluRIIA and GluRIIB intensities when the ambient temperature of wild-type flies was raised from 18 to 31°C for different periods until the wandering late third-instar larval stage (Fig. 5A). We found that synaptic GluRIIA levels began to rise and GluRIIB started to decrease after wild-type larvae were transferred from 18 to 31°C for 36 h, relative to controls reared continuously at 18°C (Fig. 5B). Synaptic GluRIIA levels were significantly elevated after wild-type larvae were reared at 31°C for 36 h, and rose continuously from 36 to 96 h [larvae raised at 31°C: 100±3.06 for 0 h (controls), 93.75±7.09 for 12 h (*P*=0.9316), 123±9.24 for 24 h (*P*=0.0644), 136.8±9.99 for 36 h (*P*=0.0004), 124.7±9.53 for 48 h (*P*=0.0377) and 143.1±14.96 for 96 h (*P*=0.0052), *F*_(5,202)_=7.002; Fig. 5C]. By contrast, synaptic GluRIIB levels were markedly reduced after wild-type larvae were reared at 31°C for 36 h, and decreased further from 36 to 96 h [larvae raised at 31°C: 100±1.88 for 0 h (controls), 95.69±3.99 for 12 h (*P*=0.9236), 103.5±6.53 for 24 h (*P*=0.9585), 85.51±4.43 for 36 h (*P*=0.0244), 83.32±4.58 for 48 h (*P*=0.0062) and 57.17±4.13 for 96 h (*P*<0.001), *F*_(5,202)_=9.90; Fig. 5C]. There were strong positive and negative correlations between the duration of high-temperature treatment and synaptic levels of GluR subtypes A and B, respectively (for subtype A, Pearson's *r*=0.82, *R*^2^=0.68, *P*=0.044, Fig. 5D; for subtype B, Pearson’s *r*=−0.95, *R*^2^=0.89, *P*=0.0042, Fig. 5E). These results show that opposite changes in the level of subtype A and B receptors occur during temperature-induced synaptic plasticity.

To understand whether the antagonistic balance of GluR subtypes was induced by increased presynaptic release that induces synaptic plasticity, we introduced the warmth-activated transient receptor potential channel (TRPA1) to induce neuronal activation by triggering a barrage of excitatory junctional potentials at NMJ termini (Hamada et al., 2008). Synaptic GluRIIA increased significantly, whereas GluRIIB did not change upon intermittent activation (IIA: 100±5.07 for controls and 140.3±5.68 for TRPA1-overexpressing larvae, *P*<0.001, *t*_(30)_=5.14; IIB: 100±5.55 for controls and 101.9±4.77 for TRPA1-overexpressing larvae, *P=*0.792, *t*_(30)_=0.27; Fig. S3). Thus, elevated neurotransmitter release resulted in increased synaptic subtype A receptors, but normal levels of subtype B receptors.

### Inhibiting the activity of GluR subtype A leads to imbalance of GluR subtypes towards more GluRIIA

**Fig. 6.**
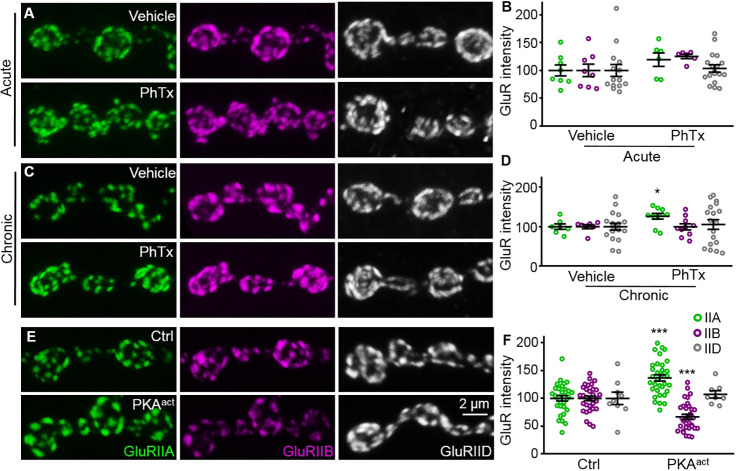
**Synaptic GluRIIA increases when the activity of GluRIIA receptors is inhibited.** (A,C,E) Representative confocal images of late third-instar larval NMJ4 stained with anti-GluRIIA (green), anti-GluRIIB (magenta) and anti-GluRIID (grey). Full genotypes and treatments are as follows: control (*C57-Gal4*/+), PKA^act^ (*UAS-PKA^act^*/+*;* *C57-Gal4*/+), vehicle (wild type treated with dimethyl sulfoxide), acute (treated with 100 μM PhTx for 30 min) and chronic PhTx treatment (treated with 5 μM PhTx from AEL until examination as late third-instar larvae). Scale bar: 2 μm in E. (B,D,F) Quantification of the intensities of anti-GluRIIA, anti-GluRIIB and anti-GluRIID staining at the NMJ. Data are expressed as normalized staining intensities with respect to vehicle treatment (B,D) and control (F). *n*≥8 for each treatment or genotype. *P<0.05; ***P<0.001. Error bars indicate s.e.m.

The response of postsynaptic cells to neurotransmitters is also involved in synaptic plasticity. To investigate whether GluR activity affected the balance of synaptic GluR subtypes, we examined synaptic GluR levels when GluRIIA activity was blocked by philanthotoxin-433 (PhTx). GluRIIA is a primary determinant of postsynaptic responses, and PhTx is an effective inhibitor of GluRIIA receptors at *Drosophila* NMJs (DiAntonio et al., 1999; Frank et al., 2006). To detect the acute effects of blocking GluRIIA, we applied 100 μM PhTx onto dissected semi-intact third-instar larvae for 30 min, following a previous protocol (Frank et al., 2006). After PhTx application, GluRIIA, GluRIIB and GluRIID remained unchanged at NMJs compared with vehicle-treated controls (IIA: 100±9.91 for controls and 119.6±12.19 for PhTx-treated larvae, *P*=0.232, *t*_(12)_=1.26; IIB: 100±11.26 for controls and 125.4±3.9 for PhTx-treated larvae, *P*=0.086, *t*_(12)_=1.87; IID: 100±10.96 for controls and 103.7±6.51 for PhTx treated larvae, *P*=0.763, *t*_(30)_=0.31; Fig. 6A,B). Given that GluRs are stable at synapses (Rasse et al., 2005), we speculated that the PhTx treatment duration of 30 min might be too short to cause changes in the level of synaptic receptors. Thus, we fed larvae with 5 μM PhTx after egg laying (AEL) until examination of late third-instar larvae and found that synaptic GluRIIA was significantly increased, whereas GluRIIB and GluRIID were unaltered (IIA: 100±6.9 for controls and 126.3±7.19 for PhTx-treated larvae, *P*=0.023, *t*_(15)_=2.53; IIB: 100±5.13 for controls and 99.59±7.82 for PhTx-treated larvae, *P*=0.969, *t*_(15)_=0.04; IID: 100±8.47 for controls and 105.4±12.2 for PhTx treated, *P*=0.720, *t*_(34)_=0.36; Fig. 6C,D). The long-term PhTx treatment results show that GluRIIA activity regulates the ratio of GluR subtypes.

To dissect the potential role of GluR activity in the balance of GluR subtypes, we took a genetic approach. Constitutively active PKA (PKA^act^) inhibits GluR largely by reducing the sensitivity of GluRIIA to glutamate (Davis et al., 1998; Sulkowski et al., 2014). Synaptic GluRIIA displayed significant accumulation, whereas GluRIIB was reduced at NMJ synapses when constitutively active PKA was expressed postsynaptically under the control of *C57-Gal4* (IIA: 100±4.91 for controls and 136.7±5.55 for PKA^act^ larvae, *P*<0.001, *t*_(62)_=4.93; IIB: 100±4.27 for controls and 66.83±4.52 for PKA^act^ larvae, *P*<0.001, *t*_(62)_=5.32; Fig. 6E,F). However, although GluRIIA and GluRIIB levels were altered, synaptic GluRIID, which represents total GluRs, remained unchanged (100±11.27 for controls and 107.4±6.56 for PKA^act^ larvae, *P*=0.593, *t*_(15)_=0.55; Fig. 6E,F). These results demonstrate that the opposite changes in subtype A and B are induced by active PKA. Altogether, we conclude that inhibition of the activity of GluR subtype A leads to imbalance of GluR subtypes towards more GluRIIA.

### Mutation of *dnc* results in an antagonistic balance of the two GluR subtypes

As presented above, the antagonistic balance between GluR subtypes is involved in temperature-induced plasticity and GluR activity-associated plasticity (Figs 5, 6). To identify genes that participate in regulation of the balance of the two GluR subtypes, we carried out a candidate screen by crossing flies with RNAi of selected genes with flies with muscle-specific *C57-Gal4*. The genes we screened were those that control GluR expression, localization or stability; we also examined *Drosophila* homologues of mammalian genes encoding GluR-associated proteins (Sigrist et al., 2000; Chen and Featherstone, 2005; Chen et al., 2005; Liebl and Featherstone, 2005, 2008; Heckscher et al., 2007; Penney et al., 2012; Lee et al., 2013). Among the genes regulating synaptic levels of either GluRIIA or GluRIIB, only *dnc* was found to induce the negative regulation of GluR subtypes (Table 1), whereas the other genes did not affect the synaptic expression of either of the two GluR subtypes (Table S1).

**Table 1.**
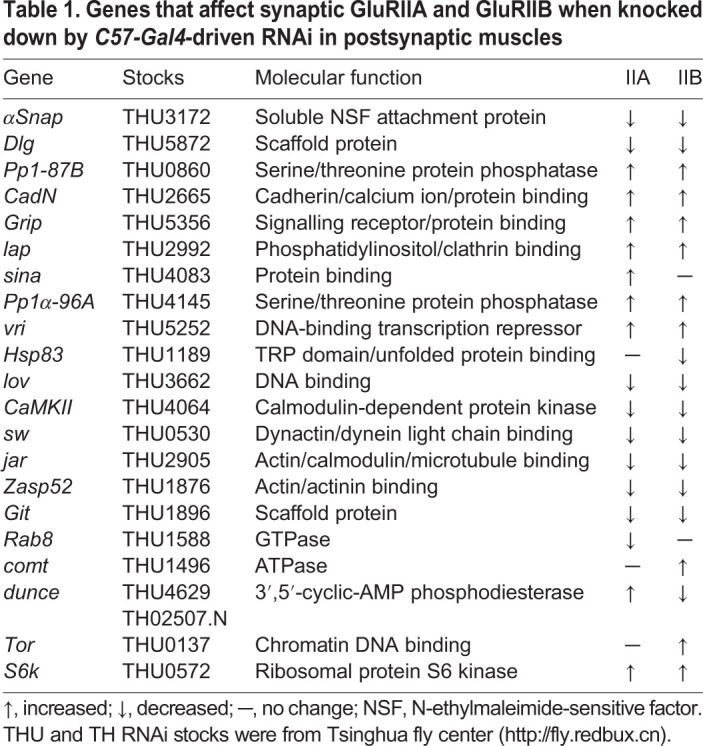
**Genes that affect synaptic GluRIIA and GluRIIB when knocked down by *C57-Gal4*-driven RNAi in postsynaptic muscles**

**Fig. 7.**
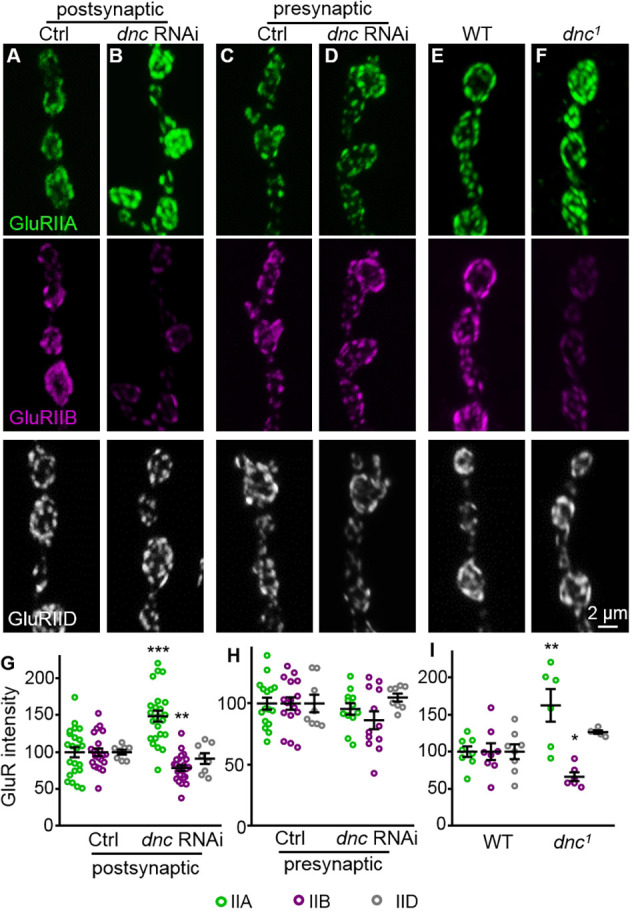
**Postsynaptic *dnc* knockdown increases GluRIIA but reduces GluRIIB levels at NMJ synapses.** (A–F) Representative images of NMJ4 synapses from different genotypes stained with anti-GluRIIA (green), anti-GluRIIB (magenta) and anti-GluRIID (grey): postsynaptic Ctrl (*C57-Gal4*/+, A), *dnc* RNAi (*C57-Gal4/UAS-dnc-RNAi*, B), presynaptic Ctrl (*OK6-Gal4*/+, C), *dnc* RNAi (*OK6-Gal4/UAS-dnc-RNAi*, D), wild type (*w^1118^*, E) and *dnc^1^* mutants (F). Scale bar: 2 μm in F. (G–I) Normalized intensities of the three GluR subunits at NMJ synapses from different genotypes. *n*=24 and *n*=16 for the genotypes shown in G and H, respectively. *n*=8 for both wild type and *dnc^1^* in I. *P<0.05; **P<0.01; ***P<0.001. Error bars indicate s.e.m.

*dnc* mutants have been shown to exhibit learning and memory defects and impaired synaptic potentiation (Zhong and Wu, 1991; Zhong et al., 1992). Herein, we found that postsynaptic knockdown of *dnc* driven by *C57-Gal4* with two independent RNAi lines (THU02507.N and THU4629, both exhibiting similar phenotypes, but only results for THU4629 are presented) induced synaptic accumulation of GluRIIA (100±6.94 for controls and 148.7±7.43 for *dnc* RNAi flies, *P*<0.001, *t*_(44)_=4.77), but a reduction of GluRIIB (100±5.03 for controls and 78.41±3.73 for *dnc* RNAi flies, *P=*0.0011, *t*_(44)_=3.48). Synaptic levels of GluRIID remained unchanged (100±3.33 for controls and 91.32±7.25 for *dnc* RNAi flies, *P=*0.295, *t*_(14)_=1.09; Fig. 7A,B,G). These RNAi results were confirmed in *dnc^1^*, a hypomorphic mutant of *dnc* (IIA: 100±7.07 for wild type and 162.7±21.85 for *dnc^1^* mutants, *P=*0.0096, *t*_(12)_=3.08; IIB: 100±11.36 for wild type and 66.14±5.91 for *dnc^1^* mutants, *P=*0.034, *t*_(12)_=2.39; IID: 100±10.03 for wild type and 126.6±2.26 for *dnc^1^* mutants, *P=*0.066, *t*_(11)_=2.04; Fig. 7E,F,I). To explore whether *dnc* acts specifically on the postsynaptic side in regulating GluRs, we examined the effect of presynaptic *dnc* reduction on GluR subtypes. Presynaptic RNAi knockdown of *dnc* by the motoneuron-specific *OK6-Gal4* resulted in normal levels of GluR subtypes, similar to controls (IIA: 100±4.83 for controls and 95.56±4.57 for *dnc* RNAi flies, *P*=0.522, *t*_(26)_=0.65; IIB: 100±5.10 for controls and 86.37±7.30 for *dnc* RNAi flies, *P*=0.126, *t*_(26)_=1.58; IID: 100±7.15 for controls and 104.9±3.27 for *dnc* RNAi flies, *P=*0.542, *t*_(14)_=0.63; Fig. 7C,D,H). Taken together, these results show that postsynaptic knockdown of *dnc* specifically elevates GluRIIA but diminishes GluRIIB at NMJ synapses.

### Bidirectional changes in postsynaptic cAMP levels result in the antagonistic balance of GluR subtypes

**Fig. 8.**
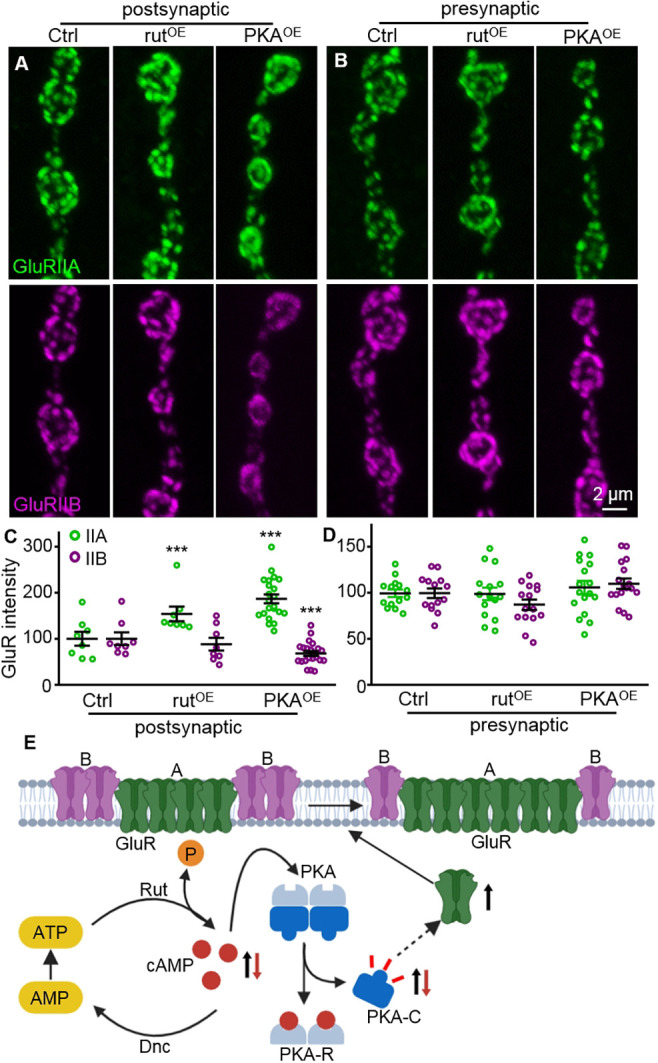
**cAMP upregulation results in increased GluRIIA but reduced GluRIIB at NMJ synapses.** (A,B) Representative images of NMJ4 synapses co-stained for GluRIIA (green) and GluRIIB (magenta) from different genotypes with postsynaptic (A) and presynaptic (B) overexpression of *rut* and *PKA*. Postsynaptic Ctrl indicates *C57-Gal4*/+, whereas presynaptic Ctrl denotes *OK6-Gal4*/+. Scale bar: 2 μm in B. (C,D) Normalized fluorescence intensities of GluRIIA and GluRIIB in different genotypes. *n*≥8 for each genotype in C; *n*≥15 for each genotype in D. ***P<0.001. Error bars indicate s.e.m. (E) Diagram depicting the role of cAMP signalling pathway in promoting the insertion of GluRIIA at PSD via PKA. PKA regulates both the protein level and the activity of GluRIIA. The step indicated by the arrow with a dashed line is not fully understood. A, GluR subtype A; B, GluR subtype B; PKA-C, activated catalytic subunit of PKA; PKA-R, regulatory subunit of PKA.

The *dnc* gene encodes phosphodiesterase II, an enzyme that hydrolyses cAMP. Hence, mutations of *dnc* lead to elevated cAMP levels (Zhong and Wu, 1991; Zhong et al., 1992). Rutabaga (Rut) is a type I adenylate cyclase that mediates cAMP synthesis, and cAMP levels are reduced in *rut* mutants (Davis, 2005). To verify a possible role for elevated cAMP in the negative regulation of GluRIIA and GluRIIB, as observed in *dnc* mutants, we examined the synaptic levels of GluRIIA and GluRIIB in larvae overexpressing Rut in postsynaptic muscles under the control of *C57-Gal4*. Compared with genetic controls, GluRIIA levels were increased dramatically, whereas GluRIIB levels were decreased slightly, without a significant difference in *rut-*overexpressing larvae (IIA: 100±5.62 for controls and 153.7±15.81 for *rut^OE^* flies, *P*=0.0003, *t*_(38)_=3.93; IIB: 100±5.55 for controls and 87.95±13.75 for *rut^OE^* flies, *P*=0.359, *t*_(38)_=0.93; Fig. 8A,C).

Protein kinase A, which is activated by cAMP, phosphorylates and activates its target proteins. To validate the effect of cAMP elevation on GluR subtype regulation, we overexpressed the catalytic subunit of PKA (PKA-C1) in postsynaptic muscles under the control of *C57-Gal4* and observed an increase in GluRIIA, but a decrease in GluRIIB at NMJ synapses (IIA: 100±5.62 for controls and 186.5±9.63 for *PKA^OE^* flies, *P*<0.001, *t*_(52)_=8.26; IIB: 100±5.55 for controls and 67.81±5.36 for *PKA^OE^* flies, *P*=0.0002, *t*_(52)_=4.01; Fig. 8A,C), similar to the effect of overexpressing constitutively active PKA (Fig. 6).

To determine whether negative regulation of GluR subtypes occurred specifically when the postsynaptic cAMP pathway was upregulated, we also analysed the synaptic levels of GluRIIA and GluRIIB when *rut* or *PKA* was overexpressed in presynaptic neurons under the control of *OK6-Gal4*. As with presynaptic *dnc* knockdown, presynaptic overexpression of Rut or PKA failed to alter the levels of GluRIIA and GluRIIB relative to controls (IIA: 100±5.23 for controls; 99.27±6.87 for *Rut^OE^* flies, *P*=0.9954; 106.2±7.12 for *PKA^OE^* flies, *P*=0.7018; *F*_(2,56)_=0.34; IIB: 100±5.07 for controls; 87.51±5.60 for *Rut^OE^* flies, *P*=0.2097; 110.3±5.60 for *PKA^OE^* flies, *P*=0.3067; *F*_(2,56)_=3.50; Fig. 8B,D). These results support the hypothesis that the cAMP-PKA pathway on the postsynaptic side specifically induces the antagonistic balance of GluR subtypes A and B, i.e. increased GluRIIA but decreased GluRIIB.

To gain a deeper understanding of the effect of cAMP on the balance of GluR subtypes, we examined synaptic GluR levels in *rut* null mutant (*rut^1^*), in which cAMP is reduced (Davis, 2005). Synaptic GluRIIA was increased significantly in *rut^1^* mutants compared with wild-type controls (100±5.78 for wild type and 241.2±18.68 for *rut^1^* mutants, *P*<0.001, *t*_(45)_=7.09; Fig. S4C,D,F). By contrast, GluRIIB was slightly, albeit significantly decreased (100±3.71 for wild type and 82±7.16 for *rut^1^* mutants, *P=*0.0329, *t*_(45)_=2.20; Fig. S4C,D,F). As expected, there was no significant difference in total GluR abundance indicated by GluRIID staining (100±6.27 for wild type and 89.34±5.28 for *rut^1^* mutants, *P=*0.2001, *t*_(46)_=1.3; Fig. S4C,D,F).

To determine whether GluR phenotypes in *rut^1^* mutants arose from postsynaptic reduction of cAMP, we examined the synaptic levels of GluRIIA, GluRIIB and GluRIID upon postsynaptic knockdown of *rut* driven by *C57-Gal4* (two RNAi lines, THU2421 and BDSC80468, were tested, and both showed similar phenotypes, but only the results for THU2421 are presented). GluRIIA levels were significantly increased compared with genetic controls (100±3.05 for controls and 118.3±4.86 for *rut* RNAi flies, *P=*0.0022, *t*_(108)_=3.133; Fig. S4A,B,E), whereas GluRIIB and GluRIID levels remained normal (IIB: 100±4.77 for controls and 104.3±5.26 for *rut* RNAi flies, *P=*0.5484, *t*_(59)_=0.6036; IID: 100±4.50 for controls and 111.4±10.41 for *rut* RNAi flies, *P=*0.3189, *t*_(46)_=1.008; Fig. S4A,B,E).

Together, these results showed that increased GluRIIA but decreased or normal GluRIIB was induced by bidirectional alterations of cAMP levels specifically in postsynaptic muscles.

## DISCUSSION

A negative correlation between subtype A and B receptors has been reported previously at *Drosophila* NMJs (Marrus et al., 2004; Pan and Broadie, 2007; Sulkowski et al., 2014). However, the mechanism by which the antagonistic balance of different subtypes of GluRs is regulated remains unclear. In the present study, we revealed that bidirectional alterations of cAMP levels in the postsynaptic muscle cells alter the balance of GluR subtypes in a cell-autonomous manner (Fig. 8E). Our study thus provides new insights into the mechanism underlying synaptic plasticity by altering the balance of GluR subtypes.

### Two GluR subtypes are distinctly localized at postsynaptic densities

Most previous conventional microscopy studies have reported substantial colocalization or differential localization of GluRIIA and GluRIIB (Marrus et al., 2004; Schmid et al., 2008; Owald et al., 2012). Herein, we report an apparently non-overlapping localization of GluRIIA and GluRIIB at the postsynaptic densities (PSDs) of NMJ synapses (Fig. 2). Although we have no clear interpretations for the distinct localization of GluRIIA and GluRIIB at PSDs, there could be two possibilities: either different classes of receptors might be associated with specific interacting proteins that could mediate, directly or indirectly, the concentric localization of GluR subtypes at PSDs (Chen et al., 2005; Wang et al., 2010) or concentric rings of GluR subtypes A and B in wild-type larvae might associate with their specific biophysical properties. Desensitization is the process by which receptors are inactivated in the prolonged presence of an agonist; it occurs faster in response to a lower concentration of agonist (Huganir and Greengardt, 1990; Heckmann and Dubel, 1997). On the postsynaptic side, GluR subtype A exhibits slower desensitization kinetics than GluR subtype B (DiAntonio et al., 1999). We therefore speculate that the slower desensitization of subtype A receptors might be caused, in part, by a higher concentration of glutamate released on the presynaptic side, because subtype A rings are more closely juxtaposed to presynaptic Cacophony calcium channels than subtype B rings (Fig. 2).

We showed that subtype A rings become enlarged (both the inner and outer ring diameters are increased) when the synaptic levels of GluRIIA are increased, whereas subtype B rings are enlarged in a specific manner, i.e. the inner diameter decreases, but the outer diameter of the ring increases when the level of GluRIIB is increased (Fig. 3). A simple explanation for the enlarged GluR rings might therefore be increased synaptic levels of GluRIIA or GluRIIB. Given that GluR-enriched PSDs are confined to specific spatial domains by cell adhesion molecules and the spectrin-actin network (Pielage et al., 2006; Hong et al., 2020), it is possible that an increase in the level of one subtype of GluR might take up the space left by a reduced level of the other.

### Alteration of the GluR subtype balance is involved in synaptic plasticity

Synaptic plasticity is the ability of synapses to strengthen or weaken over time, in response to increases or decreases in their activity. It is well established that GluRs are involved in synaptic plasticity at excitatory synapses. However, it is not entirely known how different types or subtypes of GluRs are involved in synaptic plasticity. *Drosophila* glutamatergic NMJs with two subtypes of GluRs, rather than mammalian NMJs with multiple subtypes of GluRs, are an effective model for studying synaptic plasticity. Hyperexcitable double mutants of *eag sh* show persistent strengthening of larval NMJs, which represents long-term plasticity (Budnik et al., 1990). Herein, we found that increased presynaptic release by warm-activated TrpA1 led to increased GluRIIA but normal GluRIIB (Fig. S3), which was consistent with increased GluRIIA in *eag sh* double mutants (Sigrist et al., 2000). In addition, we observed increased GluRIIA but decreased GluRIIB in high temperature-induced synaptic plasticity of long-term strengthening of neurotransmission (Sigrist et al., 2003) (Fig. 5). Thus, we speculate that increased presynaptic release might result in long-term plasticity by enhancing postsynaptic responses through increased GluRIIA.

We consistently observed increased GluRIIA in different models of synaptic plasticity. However, we observed normal and reduced GluRIIB in TrpA1- and high temperature-induced synaptic plasticity, respectively. The discrepant changes in GluRIIB levels in different models of synaptic plasticity could be caused by different timescales, such as a limited time (8 h) for elevating presynaptic release through activating TRPA1 versus 4 days for raising larvae at 27°C, or the change in the level of GluRIIB might be too low to be detected by overexpressing *TRPA1*, or both.

Whether the antagonistic balance of GluRs is actively (as a functional requirement) or passively (as a physical competition) regulated depends on specific conditions. It appeared that GluRIIA and GluRIIB competed with each other for the essential subunits when the expression levels of either GluRIIA or GluRIIB were changed (Fig. 1), consistent with previous reports (Marrus et al., 2004; Sulkowski et al., 2014). These results support a passive competition between GluRIIA and GluRIIB. However, an actively regulated antagonistic balance of GluRs also occurs (Fig. S8). When the essential subunit GluRIIC, GluRIID or GluRIIE was limited, both GluRIIA and GluRIIB decreased. If only passive regulation of GluRIIA and GluRIIB occurs, we would expect to see GluR subtype A and B decreased at similar levels. However, the ratio of GluRIIA to GluRIIB increased, indicating that the GluRIIA subtype is maintained preferentially when the total GluRs are limited (Fig. S8) and supporting an active regulation of the balance between GluRIIA and GluRIIB. Given that GluRIIA is mainly responsible for the postsynaptic responses (Petersen et al., 1997), the relative increase in GluRIIA when an essential subunit of GluRs was knocked down might be a functional compensation for the decrease of synaptic strength.

We add that in addition to the antagonism of GluRIIA and GluRIIB we report herein, there are a few reports on the regulation of synaptic levels of single GluR subunits. For example, GluRIIA but not GluRIIB receptors are anchored at the PSD by the actin-associated Coracle (Chen et al., 2005) and are regulated by a signalling pathway involving the Rho-type GEF (Pix) and its effector, Pak kinase (Albin and Davis, 2004). Our recent studies also showed specific upregulation of GluRIIA but not GluRIIB when the calcium-dependent proteinase calpains were mutated (Metwally et al., 2019).

A previous study showed that the numbers of terminal varicosities and branches were increased in *dnc* but not *rut* mutants (Zhong et al., 1992). Given that elevated cAMP levels induced an antagonistic balance of GluRs at the postsynaptic side, it was important to test whether the antagonistic balance of GluRs was associated with NMJ overgrowth. Our results showed that the number of varicosities remained normal when *dnc* or *rut* was knocked down by RNAi in the postsynaptic muscles (Fig. S6), suggesting that an alteration in the cAMP pathway at the postsynaptic side did not affect NMJ development.

The importance of the GluR subtype balance in synaptic plasticity has been documented in mammals. The major forms of AMPA receptors in the hippocampus include GluA1/2 and GluA2/3 heteromers, in addition to GluA1 homomers (Huganir and Nicoll, 2013). The relative abundance of GluA1- and GluA2-containing receptors is a well-established determinant of synaptic plasticity in diverse brain circuits; GluA1-containing receptors are recruited to synapses after long-term potentiation, whereas GluA2-containing receptors are required for long-term depression (Shi et al., 2001; Huganir and Nicoll, 2013). Together with the mammalian findings, our results support the notion that the GluR subtype balance contributes to synaptic plasticity at excitatory synapses.

### The postsynaptic cAMP pathway regulates the balance of GluR subtypes

It is widely known that cAMP signalling plays an important role in regulating synaptic plasticity by increasing presynaptic neurotransmitter release (Kandel, 2001). However, it is not known whether the cAMP pathway acts postsynaptically in regulating the ratio of GluRs, which plays a crucial role in synaptic plasticity. In the present study, we showed, for the first time, that the cAMP pathway regulates the balance of different GluR subtypes on the postsynaptic side; either increased or reduced cAMP leads to an altered ratio of GluR subtypes at *Drosophila* NMJ synapses (Figs 7, 8, S4, S5). Thus, an optimal level of cAMP in postsynaptic muscles might be required for the normal ratio of synaptic GluR subtypes.

When cAMP levels are elevated, cAMP binds to the regulatory subunits of PKA and liberates catalytic subunits that then become active (Taylor et al., 2012). Active PKA in muscles decreases the activity of GluRIIA in *Drosophila* (Davis et al., 1998). Thus, an increase in the level of synaptic GluRIIA might compensate for the reduced activity of GluRIIA caused by overexpression of wild-type or constitutively active PKA (Figs 6, 8). Conversely, inhibition of PKA activity in muscles causes a significant increase in the average amplitude of miniature excitatory junctional currents (Davis et al., 1998), consistent with the notion that PKA negatively regulates the activity of GluRIIA. Surprisingly, we also observed an increase in synaptic GluRIIA when the cAMP level was downregulated in *rut* mutants (Fig. S4) or when PKA was knocked down by RNAi (Fig. S5). It appears that the negative regulation of GluRIIA activity by PKA is not sufficient to account for the increase of GluRIIA at NMJ synapses.

Analysis of western blots showed that the protein level of GluRIIA increased significantly, regardless of whether postsynaptic cAMP pathway was up- (*dnc* RNAi and *PKA^OE^*) or downregulated (*rut* RNAi and *PKA* RNAi), suggesting that the similar antagonistic balance of GluR subtypes induced by both up- and downregulation of cAMP might be caused by an elevated protein level of GluRIIA (Fig. S7). Thus, the cAMP pathway regulates the antagonism between GluRIIA and GluRIIB at two distinct steps, GluRIIA activity and protein level. Exactly how bidirectional changes of cAMP lead to a similar alteration of GluR subtypes remains to be investigated.

Although Dnc and Rut regulate cAMP levels in opposite directions, physiological studies in *Drosophila* have shown that activity-dependent short-term plasticity is altered in a similar manner at larval NMJs in both *dnc* and *rut* mutants (Zhong and Wu, 1991). Specifically, synaptic facilitation and post-tetanic potentiation are both weakened, indicating that the bidirectional change of cAMP signalling might result in similar abnormalities in synapse plasticity (Zhong and Wu, 1991). The mechanisms underlying synaptic facilitation and post-tetanic potentiation are exclusively presynaptic. Synaptic facilitation and post-tetanic potentiation both result from increased presynaptic calcium concentrations, leading to an enhanced release of neurotransmitters. A bell-shaped model was proposed to explain this mode of regulation, i.e. mutations in *dnc* and *rut*, which regulate cAMP levels in opposite directions, result in a similar plasticity phenotype (Kim and Wu, 1996). We propose here that the bell-shaped model might also explain a similar increase in GluRIIA at NMJ synapses caused by bidirectional changes in cAMP levels in postsynaptic muscles.

The antagonistic balance of GluRIIA and GluRIIB is induced by the postsynaptic cAMP/PKA pathway. However, whether the antagonism between GluRIIA and GluRIIB requires the cAMP/PKA pathway is unclear. We have recombined GluRIIA or GluRIIB nulls with postsynaptic RNAi knockdown of PKA (i.e. inhibition of the cAMP pathway). We note that PKA null mutants are lethal at the first larval stage (Lane and Kalderon, 1993) and thus cannot be used for the genetic interaction assay. Compared with simple null mutants of GluRIIA (or GluRIIB), PKA RNAi in the mutant background of GluRIIA (or GluRIIB) did not change the synaptic levels of GluRIIB (or GluRIIA), suggesting that the antagonistic balance of GluRIIA and GluRIIB does not require the cAMP pathway at the postsynaptic side (Fig. S9). Thus, an altered cAMP pathway leads to the antagonistic balance of GluRIIA and GluRIIB, but the antagonistic balance of GluRIIA and GluRIIB appears not to be dependent on the cAMP pathway, at least for the antagonism induced by null mutations of GluRIIA or GluRIIB, or the remaining PKA upon RNAi knockdown is sufficient to support the antagonistic balance of GluRIIA and GluRIIB.

It will be of great interest to determine how the cAMP-PKA-GluR signalling pathway acts on the postsynaptic side to contribute to synaptic plasticity and whether this pathway is also effective and conserved in mammalian central synapses.

## MATERIALS AND METHODS

### *Drosophila* strains and genetics

All fly strains were reared in standard laboratory conditions at 25°C unless otherwise specified. The strain *w^1118^* was used as the wild-type control. We generated a Flag tag knock-in at the C-terminus of endogenous GluRIIB and a *GluRIIB^KO^* null allele by a CRISPR/Cas9-mediated approach largely according to previously published protocols (Port et al., 2014). The *GluRIIB* mutant carried a 381 bp DNA deletion (1906-2286 bp of sequence NT_033779.5) encoding part of the predicted glutamate-binding domain of GluRIIB and causing a frameshift mutation at 369 amino acids (AA) out of the 913 AA full-length protein.

*GluRIIA^SP16^*, *Mhc-GluRIIA*, and *Mhc-GluRIIB* were provided by A. DiAntonio (Washington University, MO, USA; Petersen et al., 1997; DiAntonio et al., 1999). *UAS-PKA^act^* was obtained from D. Kalderon (Columbia University, NY, USA; Li et al., 1995). Other stocks, including the motoneuron-specific *OK6-Gal4*, the muscle-specific *C57-Gal4*, the pan-neuronal *elav-GAL4*, *UAS-Dicer2*, *UAS-PKA-C1-Flag*, *UAS-TRPA1*, *UAS-cac1-EGFP*, *UAS-rut*, *rut* RNAi line [Bloomington *Drosophila* Stock Center (BDSC) 80468], *GluRIIC* RNAi line (BDSC 25836), *GluRIID* RNAi line (BDSC 26010), *GluRIIE* RNAi line (BDSC 25942), *rut^1^* and *dnc^1^* (a hypomorphic allele of *dnc*), were obtained from the BDSC (Indiana University, IN, USA). RNAi lines for *GluRIIA* (THU2659), *GluRIIB* (THU3089), *rut* (THU2421), *dnc* (THU4629 and THU02507.N) and *PKA-C1* (THU5744 and THU 0037) were obtained from Tsinghua Stock Center (Tsinghua University, Beijing, China).

### Immunohistochemical analyses and confocal microscopy

Immunostaining and confocal microscopy of larval preparations were performed as previously described (Xiong et al., 2013; Hong et al., 2020). Specifically, wandering third-instar larvae were dissected in Ca^2+^-free standard saline and fixed in cold methanol for 10 min on ice. The monoclonal mouse antibody against GluRIIA (1:1000; 8B4D2, concentrated form) was obtained from the Developmental Studies Hybridoma Bank (DSHB). The polyclonal rabbit antibodies against GluRIIB (1:2500) were from A. DiAntonio (Marrus et al., 2004). Rabbit anti-GluRIID (1:2500) was described previously (Qin et al., 2005). Chicken polyclonal anti-GFP (1:500; ab13970) was obtained from Abcam. Alexa 647-conjugated anti-HRP was from Jackson ImmunoResearch and used at 1:100. All primary antibodies were visualized using specific secondary antibodies conjugated with Alexa 488 or Alexa 568 (1:1000; Invitrogen). All images were obtained using an Olympus BX51 laser scanning confocal microscope and processed with ImageJ (National Institutes of Health).

Comparison of fluorescence intensities was performed as described previously (Hong et al., 2020). Mutant larval fillets were stained in the same reaction tube as genetic controls. Samples of different genotypes being compared directly were imaged at identical settings. The intensity of immunostaining was quantified as follows. A projection of the maximum immunostaining intensity at the NMJ on muscle 4 (NMJ4) was created from a series of 0.8-μm-thick sections through the entire bouton. The average fluorescence intensity was calculated over the entire synaptic area defined by HRP immunoreactivity. The fluorescence intensities of GluRs in different genotypes were normalized to the fluorescence in genetic controls.

### Structured illumination microscopy and imaging

Super-resolution images were obtained with a structured illumination microscope (Delta Vision OMX V4; GE Healthcare) as previously described (Hong et al., 2020). All images of NMJ4 from abdominal segments A2 or A3 were captured using appropriate settings for better visualization. Raw data were reconstructed to images by default reconstruction parameters of softWoRx 6.5.2 (GE Healthcare). Images were subsequently processed with Imaris 6.0 software (http://www.bitplane.com).

The inner and outer diameters of the GluR rings were measured by drawing a line through the longest diameter of the rings with ImageJ. For quantifying the numbers of GluR nanoclusters, freehand lines along the rings were drawn to trace the changes of intensities analysed with the plot profile. An intensity peak in the plot profile was counted as a GluR nanocluster.

### Real-time PCR

Quantitative real-time PCR to quantify the mRNA abundance of GluRIIA and GluRIIB was performed using the Agilent Mx3000P system (Agilent Technologies) as previously described (Jiang et al., 2016). Total mRNA was extracted from the third-instar larval muscle using an RNeasy Mini Kit (QIAGEN) and reverse transcribed using the SuperScript III First Strand Synthesis System (Invitrogen). Real-time PCR was performed concurrently for GluRIIA, GluRIIB, and actin-5C using target-specific primers. Primer sequences were as follows: sense primer 5′-CGCACCTTCACTCTGATCTATG-3′ and anti-sense primer 5′-CTGTCTCCTTCCACAATATCCG-3′ for *GluRIIA*, sense primer 5′-TCGACTCAAGCCCTTAAACAG-3′ and anti-sense primer 5′-ATTGCCCTCGTAATGGACTC-3′ for *GluRIIB*, and sense primer 5′-CAACTGGGACGATATGGAGAAG-3′ and anti-sense primer 5′-GTCTCGAACATGATCTGGGTC-3′ for *actin-5C*. The gene expression values were normalized to the expression of the *actin-5C* gene.

### Western blotting

For each genotype, 30 wandering third-instar larvae with internal organs removed were homogenized on ice in 120 μl of radioimmunoprecipitation assay buffer containing 50 mM Tris-HCl, 150 mM NaCl, 0.1% sodium dodecyl sulfate and 1% protease inhibitor mixture set I (Calbiochem). After centrifugation at 4°C for 10 min at 18,000 ***g***, the supernatant was recovered and the protein concentration determined by the Bradford assay. Equal amounts of total protein from the different genotypes were loaded onto SDS/PAGE gels. The primary antibodies used were anti-GluRIIA (1:1000; 8B4D2; DSHB), anti-Flag (1:1000; F1804; Sigma-Aldrich) and anti-α-tubulin (1:10,000; mAb B-5-1-2; Sigma-Aldrich). The primary antibodies were detected with HRP-coupled secondary antibodies (1:50,000; Sigma-Aldrich) by using a chemiluminescent method (ECL kit; Pierce). To quantify GluRIIA and GluRIIB protein levels, positive signals from multiple independent repeats were calculated using ImageJ software. GluRIIA and GluRIIB levels were normalized to the α-tubulin control.

### TRPA1 activation

Late third-instar larvae of *OK6-Gal4/UAS-TRPA1* and control (*OK6-Gal4*/+) were raised at 22°C and transferred to a PCR tube containing 50 μl of Halocarbon 700 oil (Sigma-Aldrich). Intermittent stimuli consisting of a 3 min period at a higher temperature (27°C) separated by 5 min intervals at rest (22°C) were applied in the PCR machine for 8 h based on a published protocol (Pulver et al., 2009). The larvae were then transferred to regular medium for recovery before examination by immunostaining.

### PhTx treatment of larval preparations

Semi-intact preparations were made by pinning the anterior and posterior extremities of a third-instar larva to a dissection dish, followed by a dorsal incision. For acute drug treatment, semi-intact larval preparations were incubated with PhTx (Sigma-Aldrich), an inhibitor of GluRIIA receptors (Frank et al., 2006), at a final concentration of 100 μM for 30 min. Control larvae were treated with an equal volume of vehicle containing dimethyl sulfoxide. For chronic treatment, wild-type larvae were raised in vehicle or PhTx-containing media at a final concentration of 5 μM from egg hatching. Late third-instar larvae were collected for analysis.

### Statistical analysis

All data are expressed as means±s.e.m. Statistical significance in two-way and multiple-group comparisons was determined by Student's *t-*test and one-way ANOVA with Tukey's *post hoc* test, respectively. A value of *P*<0.05 was considered statistically significant. Lack of an asterisk denotes *P*>0.05; statistical significance is denoted as *P<0.05; **P<0.01; ***P<0.001.

## Supplementary Material

Reviewer comments
