# Supplementary material for: Postsynaptic cAMP signalling regulates the antagonistic balance of Drosophila glutamate receptor subtypes
Source: Development. 2020 Dec 16;147(24):dev191874. doi: 10.1242/dev.191874 (PMC7758632; doi:10.1242/dev.191874)
Supplement: Reviewer comments [file develop-147-191874_review_history.pdf]

Postsynaptic cAMP signalling regulates the antagonistic balance of *Drosophila* glutamate receptor subtypes

Kai Zhao, Huilin Hong, Lu Zhao, Sheng Huang, Ying Gao, Elsayed Metwally, Yuqiang Jiang, Stephan J. Sigrist and Yong Q. Zhang
DOI: 10.1242/dev.191874

Editor: Francois Guillemot

Review timeline
Original submission: 1 May 2020
Editorial decision: 27 May 2020
First revision received: 26 October 2020
Accepted: 13 November 2020

Original submission

First decision letter

MS ID#: DEVELOP/2020/191874

MS TITLE: Postsynaptic cAMP signalling regulates the antagonistic balance of glutamate receptor subtypes

AUTHORS: Kai Zhao, Huilin Hong, Lu Zhao, Sheng Huang, Ying Gao, Elsayed Metwally, Yuqiang Jiang, Stephan J Sigrist, and Yong Q Zhang

I have now received the reports of three referees on your manuscript and I have reached a decision. The referees' comments are appended below, or you can access them online: please go to BenchPress and click on the 'Manuscripts with Decisions' queue in the Author Area.

As you will see, all the referees express great interest in your work, but they also have significant criticisms and recommend a substantial revision of your manuscript before we can consider publication. If you are able to revise the manuscript along the lines suggested, which may involve further experiments, I will be happy to receive a revised version of the manuscript. Your revised paper will be re-reviewed by the original referees, and its acceptance will depend on your addressing satisfactorily all their major concerns. Please also note that Development will normally permit only one round of major revision.

We are aware that you may currently be unable to access the lab to undertake experimental revisions. If it would be helpful, we encourage you to contact us to discuss your revision in greater detail. Please send us a point-by-point response indicating where you are able to address concerns raised (either experimentally or by changes to the text) and where you will not be able to do so within the normal timeframe of a revision. We will then provide further guidance. Please also note that we are happy to extend revision timeframes as necessary.

Please attend to all of the reviewers' comments and ensure that you clearly highlight all changes made in the revised manuscript. Please avoid using 'Tracked changes' in Word files as these are lost in PDF conversion. I should be grateful if you would also provide a point-by-point response detailing how you have dealt with the points raised by the reviewers in the 'Response to Reviewers' box. If you do not agree with any of their criticisms or suggestions please explain clearly why this is so.

© 2020. Published by The Company of Biologists under the terms of the Creative Commons Attribution License (https://creativecommons.org/licenses/by/4.0/).

Reviewer 1

Advance summary and potential significance to field

I agree with the authors that understanding how postsynaptic receptor fields regulate receptor subtype composition is an important and interesting question. I believe their claim that GluRIIA and GluRIIB are antagonistically regulated in terms of their relative representation in postsynaptic glutamate receptor fields - Figure 1 is compelling, showing that GluRIIA knockdown/knockout increases GluRIIB abundance (and vice versa), and that IIA overexpression increases IIA at the expense of IIB (and vice versa).

Comments for the author

The manuscript by Zhao et al. builds on prior observations in the field on GluRIIA/B antagonism to link postsynaptic PKA and cAMP levels to this biology. The authors do some nice experiments to verify these previous observations, but the amount of new information provided here is limited. However, their model depicted in figure 8 is not supported by their data – both increases and decreases in cAMP levels leads to excess GluRIIA, so it is not a simple picture as they suggest -- PKA phosphorylates GluRIIA and enhances its insertion. There are also some manipulations that do not show the IIA/B antagonism, breaking this rule and only showing increased IIA. As such, it is hard to know why the rule applies or doesn't during some manipulations. When it doesn't apply, what is going on? If the authors could answer the major question of the paper – when the levels of cAMP are downregulated, what is the mechanism increasing the GluRIIA/IIB ratio – I would be more enthusiastic. In addition, its clear some of this biology is also pKA independent, so that needs to be more clearly discussed. Finally a clearer understanding of what exactly the IIA/B antagonism means in terms of the basic biology of the whole GluR tetramer is important.

Major comments:

1. What it the underlying biology at play here. Based on the data in figure 1, a key unanswered question emerges:
Is this passive competition based on IIA/IIB availability for binding to obligatory GluR subunits (the simple model) or is this actively regulated competition (the more interesting model)?

The maintenance of total GluRIID at WT levels is consistent with a simple model where this "mutually negative IIA/IIB regulation" arises merely from passive competition between IIA and IIB subunits for assembling with obligatory subunits. If this antagonistic balance is maintained in all situations that trigger a change in either IIA or IIB abundance, that supports the simple model that the competition is passive.
Alternatively, if the negative regulation of IIA and IIB can be expressed in some manipulations but not others, that suggests a more active plasticity mechanism. For this reason, I found it interesting that increasing presynaptic release through TrpA activation (Fig S2) increased IIA without antagonistically decreasing IIB. This might suggest that the antagonistic regulation between the receptor subtypes is not merely a passive consequence of relative IIA/IIB protein availability. However, if the effect is active or passive is unclear.

2. The main conclusion of this paper is that a IIA/IIB antagonistic balance is mediated by a postsynaptic cAMP/PKA pathway. I don't feel they have enough evidence to make this claim, and I wonder if the PKA/cAMP pathway is just acting as a trigger for the antagonistic balance by regulating IIA abundance. With any manipulation in the PKA pathway that leads to opposing IIA/IIB alterations, the key experiment that distinguishes whether the pathway is mediating the antagonistic competition vs merely triggering it is as follows: you need to trigger the competition by altering IIA or IIB levels (as in figure 1) in the background of a broken postsynaptic PKA pathway and show that the pathway is actually required for the antagonistic regulation to occur. The authors didn't do this – they just showed that altering the PKA/cAMP pathway results in the antagonistic receptor balance being triggered, but they do not provide sufficient evidence to claim that this pathway mediates the balance instead of merely triggering it.

© 2020. Published by The Company of Biologists under the terms of the Creative Commons Attribution License (https://creativecommons.org/licenses/by/4.0/).

3. Figure 6 is particularly confusing. In the chronic Phtx manipulation, they don't observe antagonistic regulation despite seeing an increase in IIA levels. They are claiming that IIA activity leads to an increase in IIA and a decrease in IIB, but the only data in this figure that supports that is their PKA mutant (PKA-act). They use this mutant to manipulate GluRIIA levels, but PKA is also involved in the pathway they claim regulates the antagonistic balance. Does IIA activity initiate this IIA/IIB competition in the background of normal postsynaptic PKA activity?

4. It would be helpful to plot not only bar graphs with error bars, but also individual points, for all the figures.

5. Upon acute or chronic GluRIIA blockage by PhTx, GluRIIA increases slightly (in the chronic condition) but GluRIIB does not decrease, contrary to what the figure title says. The PKA manipulation does show opposite changes in the abundance of GluRIIA and GluRIIB, but that's not a clean inhibition of GluRIIA activity and is confusing because the authors are also claiming that PKA is responsible for mediating the antagonistic balance.

6. Do these results actually demonstrate that the cAMP pathway is mediating the antagonistic balance observed in figure 1, or could it just be regulating IIA and then IIB responds via a separate pathway? It seems like you would need to overexpress or knock down IIA or IIB in the background of a broken postsynaptic cAMP pathway and show that the antagonistic regulation requires this pathway.

Minor Comments:
1. In Figure 4B, what is going on with the two bands and why does the lower one go away upon GluRIIB RNAi but not strengthen with GluRIIA overexpression? Some discussion of the two bands would be helpful.
2. In 4D and 4E, it would be helpful to show in the same western blot that GluRIIB goes up and down with overexpression and RNAi so we know the quantitative western is in a range where changes could be detected.

<u>Reviewer 2</u>

Advance summary and potential significance to field

The data from Zhao et al., sets a baseline and basic understanding of glutamate receptor (GluR) subtype regulation at the Drosophila larval neuromuscular junction. The Drosophila larval NMJ synapse is an attractive and widely used system to study synapse development and plasticity. The GluR composition has been described over time via the efforts many labs. For some time now is has been known that the abundance of GluRIIA vs GluRIIB can vary. This work neatly describes an antagonism between the two GluRs with the GluRD subunit (which is invariant) unchanging. Increasing activity either with temperature shifts, or pre-synaptic ion channel expression sees increases in GlurIIA, while inhibiting GluRIIA sees an increase in this subunit. There has been a history of papers showing a role for cAMP regulation of properties of this synapse, mostly for measures of physiological plasticity, and this work now starts a framework for our understanding of this plasticity in terms of GluR juggling. My surprise is that for both loss-of-function for rutabaga and dunce (loss and increase of cAMP signaling) there is an increase of GluRIIA at the synapse and I struggle to reconcile this.
Nonetheless, I find the paper attractive and commendable. One measure of plasticity that is missing is the measure of synapse size with the manipulations presented (it is know that dunce and rutabaga manipulations can alter synapse size). This would be an extensive and difficult dataset to incorporate and I'm not convinced it would add to the narrative of this paper substantially. The GluR abundance has an explanatory power, whiile the reasons for synaptic growth are as yet unlcear. I would suggest the authors address this textually, or refer to data published or that they have on synapse size (incorporate into the supplementary?).

Comments for the author

I have very little to critique for this paper. It is very clean.

© 2020. Published by The Company of Biologists under the terms of the Creative Commons Attribution License (https://creativecommons.org/licenses/by/4.0/).

Anti-HRP – add source and how it was used.

C57-GAL4 not mentioned in methods.

It would be good to have the GluRD antibody control for the PhTx treatment.

I have a question about reconciling the loss and gain of function for cAMP and the increase in GluRIIA for both. In the expression of PKAact, is there a change in migration by western for GlurIIA? – is the same seen in dnc mutants or overexpression of rut? For dnc or rut LOF, GluRIIA is upregulated, when one might expect opposite effects. A western blot of GlurIIA for phosphorylation may reconcile these results – potentially teasing out three effective receptors: GlurIIB, GluRIIA and GlurIIA-Phosphorylated (where for the latter it is stated that PKA mediated phosphorylation alters the gating of the channel). I am loath to ask for further experiments, but I feel that this would help clarify what is a question that is raised by the data, but ducked in the discussion.

Reviewer 3

Advance summary and potential significance to field

The manuscript by Zhao et al describes the balance between two glutamate receptor subunits IIA and IIB in postsynapse of Drosophila neuromuscular junctions. They first performed systematic measurements of IIA or IIB levels in RNAi and overexpression of another subunit, showing negative correlations between these two subunits in postsynapses. By performing SIM super-resolution imaging, they show that IIA and IIB formed two concentric rings with the IIA ring surrounded by the IIB ring, and the ring sizes are regulated by the expression levels of the other subunit. The reciprocal regulations are not transcriptional neither at the total protein levels, suggesting that IIA/IIB balance is at postsynaptic sites, possibly receptor localization. Several manipulations including heat treatment, blocking presynaptic exocytosis, inhibiting IIA receptors, all lead to increase of IIA without significant alterations on IIB. To identify the regulation IIA/IIB balance, a small-scale genetic screen for possible candidates identified Dunce (cAMP phosphodiesterase). In dunce mutants (or later with PKA overexpression), the IIA levels are increased and the IIB levels are reduced. Since PKA pathway has been suggested to downregulate quantal size in a previous study (Davis, 1998), they propose that the increase of postsynaptic IIA levels is compensatory to the reduced quantal size.

Overall, the study is very interesting, aiming to uncover a well-known phenomenon in the field. The high-resolution images of GluRIIA and IIB localization in postsynapses (Fig 2) is very impressive, which could provide new directions of study. This is also the most improved part of the manuscript compared to a previous version I had the chance to review. Overall, I think there is significant improvement in the manuscript, which is suitable for publication with modifications as follow (mainly on data analysis and writing):

Comments for the author

1. The increase or decrease of one subunit (IIA or IIB) lead to reverse changes in another subunit, while the co-subunit level (IID) is unchanged. I wonder the negative correlation of IIA and IIB are confined by the total amount of IID available to assemble GluRs or for localization. Have authors tested whether manipulating IID levels could affect IIA and IIB levels or allow an increase of one subunit without compromising the other's level? If not, could this be discussed if it is a possible explanation?

2. It is very nice to see the better images of IIA and IIB forming concentric rings in postsynapses, with IIA taking the more central position. The morphology of the ring structures is depicted in Fig 2H, with connected four lobes for each ring. One question is that whether all IIA/IIB rings they observed are all in tetrameric forms? Are there other shapes or IIA only or IIB only rings (In particular when IIA/IIB ratios are altered)? Statistics is necessary to describe the percentages of clusters that are in tetrameric forms or in other forms. Also, the calculated lobe size (from Fig 2H) would be about 200nm in diameter, comparing to the structure of a single glutamate receptor of less than 20nm, it would be very informative to estimate the numbers of receptors in each lobe.

© 2020. Published by The Company of Biologists under the terms of the Creative Commons Attribution License (https://creativecommons.org/licenses/by/4.0/).

When the lobe size changes with increases in IIA or IIB levels, the numbers of receptors would be changed accordingly. It would be nice if the authors could estimate the “numbers” of receptors in some plasticity model and describe that in Discussion, which would provide a more quantitative view.

3. It would be nice to show the Flag-tagged IIB images (immunostained by Flag antibody) in postsynapses, to reveal how the postsynaptic localization pattern compared to IIB immunostaining. If they are comparable, it would be a nice contribution to the field to use this Flag-IIB line.

4. It appears that some part of this reciprocal regulation is at protein localization, not total protein levels, to the postsynapses. Some pathways are known to regulate IIA localization (or postsynaptic levels), which could be incorporated in the Discussion.

First revision

Author response to reviewers' comments

Reviewer 1 Advance Summary and Potential Significance to Field:
I agree with the authors that understanding how postsynaptic receptor fields regulate receptor subtype composition is an important and interesting question. I believe their claim that GluRIIA and GluRIIB are antagonistically regulated in terms of their relative representation in postsynaptic glutamate receptor fields - Figure 1 is compelling, showing that GluRIIA knockdown/knockout increases GluRIIB abundance (and vice versa), and that IIA overexpression increases IIA at the expense of IIB (and vice versa).

Reviewer 1 Comments for the Author:
The manuscript by Zhao et al. builds on prior observations in the field on GluRIIA/B antagonism to link postsynaptic PKA and cAMP levels to this biology. The authors do some nice experiments to verify these previous observations, but the amount of new information provided here is limited. However, their model depicted in figure 8 is not supported by their data – both increases and decreases in cAMP levels leads to excess GluRIIA, so it is not a simple picture as they suggest -- PKA phosphorylates GluRIIA and enhances its insertion. There are also some manipulations that do not show the IIA/B antagonism, breaking this rule and only showing increased IIA. As such, it is hard to know why the rule applies or doesn’t during some manipulations. When it doesn’t apply, what is going on? If the authors could answer the major question of the paper – when the levels of cAMP are downregulated, what is the mechanism increasing the GluRIIA/IIB ratio – I would be more enthusiastic. In addition, its clear some of this biology is also pKA independent, so that needs to be more clearly discussed. Finally a clearer understanding of what exactly the IIA/B antagonism means in terms of the basic biology of the whole GluR tetramer is important.

Reply: Very good comments and suggestions! We have added to the model that a decrease in cAMP level and PKA activity also leads to the antagonistic balance of GluRs at NMJ synapses (Fig. 8E).

Our results demonstrate that the antagonistic balance of GluR subtypes occurs under certain conditions, particularly under high-temperature-induced plasticityshown to be mediated by the increased motoneuron action potential frequencies associated (Sigrist et al., 2003; Fig. 5) and the cAMP-PKA pathway-mediated plasticity (Figs. 7,8,S4,S5). In support of our results, a few proteins are also known to regulate the antagonistic balance of GluR subtypes. For example, the translational repressor Pumilio inhibits GluRIIA expression and promotes GluRIIB expression through inhibition of elF4E (Sigrist et al., 2000; Menon et al., 2004). However, Nanos, a corepressor of Pumilio, acts in opposition to Pumilio in regulating the subunit composition of the glutamate receptors (Menon et al., 2009). In *Drosophila fragile X mental retardation 1* (*dfmr1*) null mutants, GluR subtype A accumulates while GluR subtype B is decreased, whereas the total GluR levels do not change (Pan and Broadie, 2007). The exact role of GluRIIA/IIB antagonism in synaptic plasticity and function remains to be further elucidated.

© 2020. Published by The Company of Biologists under the terms of the Creative Commons Attribution License (https://creativecommons.org/licenses/by/4.0/).

We do not know the exact mechanism of increased GluRIIA/IIB ratio when the level of cAMP is downregulated. However, we found that the protein level of GluRIIA increased significantly when cAMP was downregulated, suggesting that the increased protein levels of GluRIIA might be a reason for the increased ratio of GluRIIA/GluRIIB (for details, please see the replies to the comment 4 of reviewer 2).

Our results show that altered cAMP pathway leads to the antagonistic balance of GluRIIA/IIB, but the antagonistic balance of GluRIIA/IIB, at least for the GluRIIA or GluRIIB null-induced balance, appears not dependent on the cAMP pathway (for details, please see the replies to the comment 2 below).

Major comments:

1. What it the underlying biology at play here. Based on the data in figure 1, a key unanswered question emerges: Is this passive competition based on IIA/IIB availability for binding to obligatory GluR subunits (the simple model) or is this actively regulated competition (the more interesting model)?

The maintenance of total GluRIID at WT levels is consistent with a simple model where this "mutually negative IIA/IIB regulation" arises merely from passive competition between IIA and IIB subunits for assembling with obligatory subunits. If this antagonistic balance is maintained in all situations that trigger a change in either IIA or IIB abundance, that supports the simple model that the competition is passive. Alternatively, if the negative regulation of IIA and IIB can be expressed in some manipulations but not others, that suggests a more active plasticity mechanism. For this reason, I found it interesting that increasing presynaptic release through TrpA activation (Fig S2) increased IIA without antagonistically decreasing IIB. This might suggest that the antagonistic regulation between the receptor subtypes is not merely a passive consequence of relative IIA/IIB protein availability. However, if the effect is active or passive is unclear.

Reply: Whether the antagonistic balance of GluRs is actively (as a functional requirement) or passively (as a physical competition) regulated depends on specific conditions. It appears that GluRIIA and GluRIIB compete with each other for the essential subunits when the expression levels of either GluRIIA or GluRIIB was changed (Fig. 1), consistent with previous reports (Marrus et al., 2004; Sulkowski et al., 2014). These results support a passive competition between GluRIIA and GluRIIB. However, an actively regulated antagonistic balance of GluRs also occurs (see replies to comment 1 of the reviewer 3; Fig S8). When the essential subunit GluRIIC, GluRIID, or GluRIIE was limited, both GluRIIA and GluRIIB decreased. If only passive regulation of GluRIIA and GluRIIB occurs, we would expect to see GluR subtype A and B decreased at similar levels. However, the ratio of GluRIIA/GluRIIB increased, indicating that the GluRIIA subtype is preferentially maintained when the total GluRs are limited (see replies to comment 1 of the reviewer 3; Fig S8), supporting an active regulation of GluRIIA/IIB balance. We have added this form of active regulation of GluRIIA and GluRIIB in the Discussion in lines 562 to 577.

2. The main conclusion of this paper is that an IIA/IIB antagonistic balance is mediated by a postsynaptic cAMP/PKA pathway. I don't feel they have enough evidence to make this claim, and I wonder if the PKA/cAMP pathway is just acting as a trigger for the antagonistic balance by regulating IIA abundance. With any manipulation in the PKA pathway that leads to opposing IIA/IIB alterations, the key experiment that distinguishes whether the pathway is mediating the antagonistic competition vs merely triggering it is as follows: you need to trigger the competition by altering IIA or IIB levels (as in figure 1) in the background of a broken postsynaptic PKA pathway and show that the pathway is actually required for the antagonistic regulation to occur. The authors didn't do this - they just showed that altering the PKA/cAMP pathway results in the antagonistic receptor balance being triggered, but they do not provide sufficient evidence to claim that this pathway mediates the balance instead of merely triggering it.

Reply: A very good suggestion! We have recombined GluRIIA or GluRIIB nulls with postsynaptic RNAi knockdown of PKA (i.e., inhibition of cAMP pathway). We note that PKA null mutants are lethal at 1st larval stage (Lane and Kalderon, 1993) and thus cannot be used for the genetic interaction assay. Compared with simple null mutants of GluRIIA (or GluRIIB), PKA RNAi in the mutant background of GluRIIA (or GluRIIB) did not change the synaptic levels of GluRIIB (or GluRIIA) (see figure below), suggesting that the antagonistic balance of GluRIIA/IIB does not require the cAMP pathway at the

© 2020. Published by The Company of Biologists under the terms of the Creative Commons Attribution License (https://creativecommons.org/licenses/by/4.0/).

postsynaptic side. Thus, an altered cAMP pathway leads to the antagonistic balance of GluRIIA/IIB, but the antagonistic balance of GluRIIA/IIB appears not dependent on the cAMP pathway, at least for the antagonism induced by null mutations of GluRIIA or GluRIIB, or the remaining PKA upon RNAi knockdown is sufficient to support the antagonistic balance of GluRIIA/IIB. We have added this point with a new figure (Fig. S9, also shown below) in Discussion in lines 646 to 659.

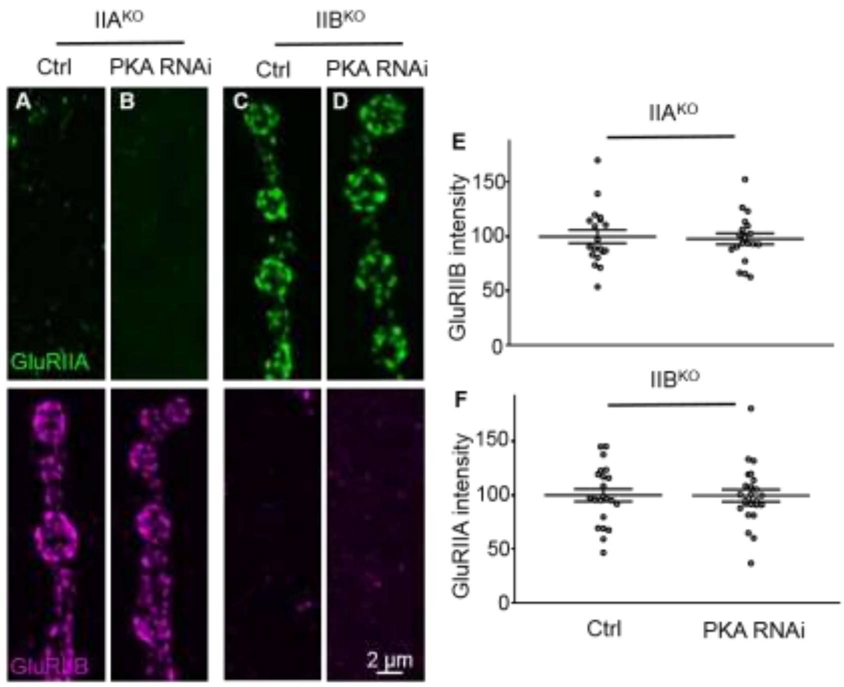

Fig. S9. The antagonistic balance of GluRIIA/IIB does not require the cAMP pathway on the postsynaptic side. (A-D) Representative images of NMJ4 synapses from different genotypes stained with anti-GluRIIA (green) and anti-GluRIIB (magenta): IIA^{KO} Ctrl (*IIA^{SP16}; C57-Gal4/+*, A), IIA^{KO} PKA RNAi (*IIA^{SP16}; C57-Gal4/UAS-PKA RNAi*, B), IIB^{KO} Ctrl (*IIB^{KO}; C57-Gal4/+*, C), and IIB^{KO} PKA RNAi (*IIB^{KO}; C57-Gal4/UAS-PKA RNAi*, D). Scale bar: 2 µm. (D,E) Normalized intensities of GluRIIB and GluRIIA at NMJ synapses from different genotypes. $n \geq 19$ for each genotype. Error bars indicate the s.e.m.

3. Figure 6 is particularly confusing. In the chronic Phtx manipulation, they don't observe antagonistic regulation despite seeing an increase in IIA levels. They are claiming that IIA activity leads to an increase in IIA and a decrease in IIB, but the only data in this figure that supports that is their PKA mutant (PKA-act). They use this mutant to manipulate GluRIIA levels, but PKA is also involved in the pathway they claim regulates the antagonistic balance. Does IIA activity initiate this IIA/IIB competition in the background of normal postsynaptic PKA activity?

Reply: This point is similar to the point 5 below (please see the replies to the point 5 below). Our results show that decreased GluRIIA activity results in the imbalance of GluR subtypes towards more GluRIIA in the background of normal postsynaptic PKA activity (Fig. 6).

4. It would be helpful to plot not only bar graphs with error bars, but also individual points, for all the figures.

Reply: As suggested, to better illustrate the original data points, we have changed bar graphs to scatter plot graphs.

5. Upon acute or chronic GluRIIA blockage by PhTx, GluRIIA increases slightly (in the chronic condition) but GluRIIB does not decrease, contrary to what the figure title says. The PKA manipulation does show opposite changes in the abundance of GluRIIA and GluRIIB, but that's not a clean inhibition of GluRIIA activity and is confusing because the authors are also claiming that PKA is responsible for mediating the antagonistic balance.

© 2020. Published by The Company of Biologists under the terms of the Creative Commons Attribution License (https://creativecommons.org/licenses/by/4.0/).

Reply: Active PKA is used as an inhibitor of GluRIIA in the community (Davis et al., 1998; Sulkowki et al., 2014). Postsynaptic expression of active PKA decreases quantal size, while the reduction of quantal size by active PKA is lost in *GluRIIA* null mutants (Davis et al., 1998). We thus expressed active PKA in postsynaptic muscles to examine its effect on the balance of GluIIA and GluRIIB. To better understand the changes of GluRIIA/IIB upon PhTx treatment, we stained with anti-GluRIID upon both acute and chronic PhTx treatment (also see replies to comment 3 of reviewer 2) and found normal synaptic GluRIID (Fig. 6C,D). We speculate that an antagonistic balance between GluRIIA and GluRIIB might still exist after chronic PhTx treatment, as a moderately elevated level of GluRIIA might not lead to a significant decrease in GluRIIB (Fig. 6C,D). Thus, we toned down the statement that inhibiting the activity of GluR subtype A leads to imbalance of GluR subtypes towards more GluRIIA. We added the detailed results in lines 369 to 406. We made corresponding modifications on results, figure, and figure legends associated with Fig. 6 C and D.

6. Do these results actually demonstrate that the cAMP pathway is mediating the antagonistic balance observed in figure 1, or could it just be regulating IIA and then IIB responds via a separate pathway? It seems like you would need to overexpress or knock down IIA or IIB in the background of a broken postsynaptic cAMP pathway and show that the antagonistic regulation requires this pathway.

Reply: A similar question as point 2 (please see the replies to point 2 above). Our results show that the antagonistic balance of GluRIIA/IIB, at least for the GluRIIA or GluRIIB null-induced balance, appears not dependent on the cAMP pathway (Fig. S9).

Minor Comments:

1. In Figure 4B, what is going on with the two bands and why does the lower one go away upon GluRIIB RNAi but not strengthen with GluRIIA overexpression? Some discussion of the two bands would be helpful.

Reply: The lower band might be a non-specific band recognized by anti-IIA, but off- targeted by the IIB RNAi, as the lower band did not change when GluRIIA was overexpressed or knocked down by RNAi.

2. In 4D and 4E, it would be helpful to show in the same western blot that GluRIIB goes up and down with overexpression and RNAi so we know the quantitative western is in a range where changes could be detected.

Reply: As suggested, we did anti-Flag western blotting for GluRIIB RNAi knockdown using the stock of Flag knockin at the C-terminus of the endogenous IIB. As far as we know there are no tools to overexpress IIB-Flag at present. We found that the protein level of GluRIIB detected by anti-Flag dramatically decreased when GluRIIB was knocked down by RNAi. The original Fig. 4D and Fig. 4E were replaced with the new western results below.

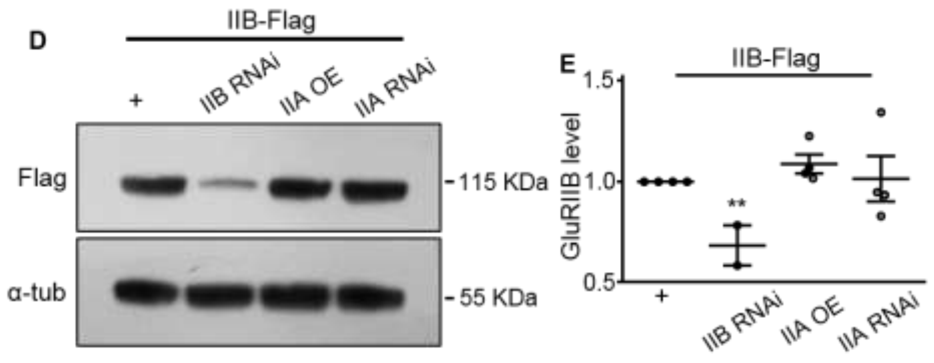

Fig. 4. Negative regulation between GluRIIA and GluRIIB occurs at post- transcriptional level.(D) Representative western blots of muscle lysates used for quantifying the total amount of Flag-tagged

© 2020. Published by The Company of Biologists under the terms of the Creative Commons Attribution License (https://creativecommons.org/licenses/by/4.0/).

GluRIIB. The full genotypes are as follows: IIB-Flag/+ (GluRIIB-Flag/+), IIB-Flag/IIB RNAi (GluRIIB-Flag/+; C57-Gal4, UAS- GluRIIB RNAi/+), IIB-Flag/IIA OE (Mhc-GluRIIA, GluRIIB-Flag/+), IIB-Flag/IIA RNAi (GluRIIB-Flag/+; C57-Gal4, UAS-GluRIIA RNAi/+). α-tubulin was used as a loading control. (E) Quantification of GluRIIB protein levels normalized to the α- tubulin control in different genotypes. n > 3. **p < 0.01. Error bars indicate s.e.m.

Reviewer 2 Advance Summary and Potential Significance to Field:
The data from Zhao et al., sets a baseline and basic understanding of glutamate receptor (GluR) subtype regulation at the Drosophila larval neuromuscular junction. The Drosophila larval NMJ synapse is an attractive and widely used system to study synapse development and plasticity. The GluR composition has been described over time via the efforts many labs. For some time now is has been known that the abundance of GluRIIA vs GluRIIB can vary. This work neatly describes an antagonism between the two GluRs with the GluRD subunit (which is invariant) unchanging. Increasing activity either with temperature shifts, or pre-synaptic ion channel expression sees increases in GlurIIA, while inhibiting GluRIIA sees an increase in this subunit. There has been a history of papers showing a role for cAMP regulation of properties of this synapse, mostly for measures of physiological plasticity, and this work now starts a framework for our understanding of this plasticity in terms of GluR juggling. My surprise is that for both loss-of-function for rutabaga and dunce (loss and increase of cAMP signaling) there is an increase of GluRIIA at the synapse and I struggle to reconcile this. Nonetheless, I find the paper attractive and commendable. One measure of plasticity that is missing is the measure of synapse size with the manipulations presented (it is know that dunce and rutabaga manipulations can alter synapse size). This would be an extensive and difficult dataset to incorporate and I'm not convinced it would add to the narrative of this paper substantially. The GluR abundance has an explanatory power, while the reasons for synaptic growth are as yet unlcear. I would suggest the authors address this textually, or refer to data published or that they have on synapse size (incorporate into the supplementary?).

Reply: A previous report shows that the numbers of terminal varicosities and branches are increased in *dnc* but not *rut* mutants (Zhong et al., 1992). We observed that the number of varicosities did not change appreciably when *dnc* or *rut* was knocked down by RNAi in postsynaptic muscles, suggesting that altered cAMP pathway at the postsynaptic side did not affect morphology of NMJs (see figure below). Wediscussed this point in Discussion with a new figure (Fig. S6, shown below) in lines 585 to 591.

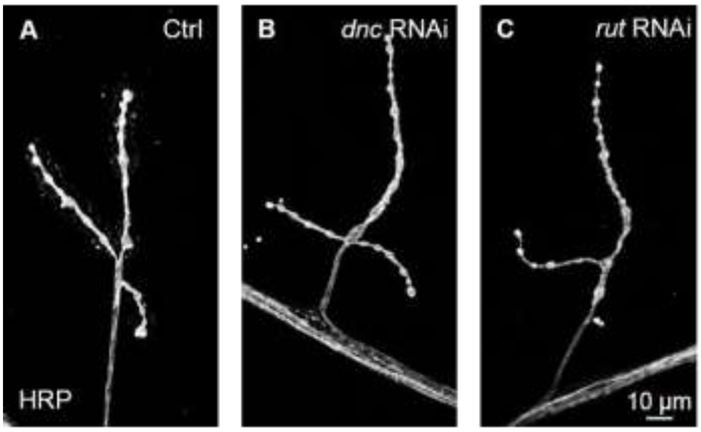

Fig. S6. The number of varicosities remains normal when *dnc* or *rut* is knocked down by RNAi in postsynaptic muscles. (A-C) Representative images of NMJ4 synapses stained with anti-HRP (gray). The genotypes are Control (Ctrl, C57-Gal4/+), *dnc* RNAi (C57-Gal4/UAS-dnc RNAi) and *rut* RNAi (C57-Gal4/UAS-rut RNAi). Scale bar: 10 μm.

Reviewer 2 Comments for the Author:

1. I have very little to critique for this paper. It is very clean. Anti-HRP – add source and how it was used.

Reply: We described the source and usage of anti-HRP in the “Immunohistochemical analyses and confocal microscopy” section of the Materials and Methods in the original manuscript. It reads that

© 2020. Published by The Company of Biologists under the terms of the Creative Commons Attribution License (https://creativecommons.org/licenses/by/4.0/).

"Alexa 647-conjugated anti-horseradish peroxidase (HRP) was from Jackson ImmunoResearch and used at 1:100". To clarify the usage of anti-HRP, we have added "We assessed the abundance of GluRIIA and GluRIIB over the entire synaptic area defined by immunostaining with antibodies against horse radish peroxidase (HRP) upon altering the expression of either GluRIIA or GluRIIB." in the Results (lines 208-210).

2. C57-GAL4 not mentioned in methods.

Reply: In *Drosophila strains and genetics* section of the Materials and Methods of the original manuscript, we described the stock *C57-Gal4*. It says that "Other stocks including the motoneuron-specific *OK6-Gal4*, the muscle specific *C57-Gal4*, ……were obtained from the Bloomington Stock Centre (Indiana University, IN)".

3. It would be good to have the GluRD antibody control for the PhTx treatment.
Reply: As suggested, we have stained GluRIID for acute and chronic treatment of PhTx and found that GluRIID remained normal upon PhTx treatments. These results have been integrated in the revised Figure 6.

4. I have a question about reconciling the loss and gain of function for cAMP and the increase in GluRIIA for both. In the expression of PKAact, is there a change in migration by western for GlurIIA? – is the same seen in dnc mutants or overexpression of rut? For dnc or rut LOF, GluRIIA is upregulated, when one might expect opposite effects. A western blot of GlurIIA for phosphorylation may reconcile these results – potentially teasing out three effective receptors: GlurIIB, GluRIIA and GlurIIA-Phosphorylated (where for the latter it is stated that PKA mediated phosphorylation alters the gating of the channel). I am loath to ask for further experiments, but I feel that this would help clarify what is a question that is raised by the data, but ducked in the discussion.

Reply: A very good suggestion! As far as we know, there is no antibody available for phosphorylated GluRIIA. We thus performed anti-GluRIIA western analysis after manipulating PKA, Dnc, Rut and found no altered migration of GluRIIA, suggesting that phosphorylation of GluRIIA by PKA might not affect the molecular weight of GluRIIA. Remarkably, we found that the protein level of GluRIIA increased significantly no matter postsynaptic cAMP pathway was up- (*dnc* RNAi and *PKA*^{OE}) or downregulated (*rut* RNAi and *PKA* RNAi; Fig S7). These results suggest that the similar antagonistic balance of GluR subtypes induced by both up and down-regulation of cAMP might be caused by the elevated protein level of GluRIIA. We discussed this point in Discussion in lines 624 to 631.
Early studies report that active PKA inhibits GluRIIA activity (Davis et al., 1998; Sulkowski et al., 2014). To our surprise, the Western results show that cAMP pathway also regulates GluRIIA protein level. Thus, cAMP pathway regulates GluRIIA/IIB antagonism at two distinct steps, GluRIIA activity and protein level.

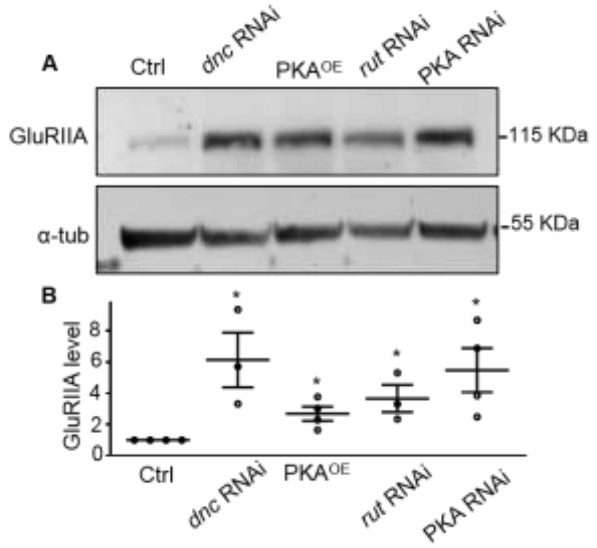

© 2020. Published by The Company of Biologists under the terms of the Creative Commons Attribution License (https://creativecommons.org/licenses/by/4.0/).

Fig. S7. The protein level of GluRIIA increases significantly when cAMP pathway is up- or down-regulated. (A) Representative western blots of muscle lysates probed with anti-GluRIIA. The full genotypes are as follows: Ctrl (*C57-Gal4/+*), *dnc* RNAi (*C57-Gal4/UAS-dnc RNAi*), PKA^{OE} (*C57-Gal4/UAS-*PKA^{OE}), *rut* RNAi (*UAS-rut-RNAi/+; C57-Gal4/+*), and *PKA-C1* RNAi (*C57-Gal4/UAS-PKA-C1-RNAi*). α-tubulin was used as a loading control. (B) Quantification of GluRIIA protein levels normalized to the α-tubulin control in different genotypes. $n \geq 3$. $^{*}p < 0.05$. Error bars indicate the s.e.m.

Reviewer 3 Advance Summary and Potential Significance to Field:
The manuscript by Zhao et al describes the balance between two glutamate receptor subunits IIA and IIB in postsynapse of Drosophila neuromuscular junctions. They first performed systematic measurements of IIA or IIB levels in RNAi and overexpression of another subunit, showing negative correlations between these two subunits in postsynapses. By performing SIM super-resolution imaging, they show that IIA and IIB formed two concentric rings with the IIA ring surrounded by the IIB ring, and the ring sizes are regulated by the expression levels of the other subunit. The reciprocal regulations are not transcriptional neither at the total protein levels, suggesting that IIA/IIB balance is at postsynaptic sites, possibly receptor localization. Several manipulations including heat treatment, blocking presynaptic exocytosis, inhibiting IIA receptors, all lead to increase of IIA without significant alterations on IIB. To identify the regulation IIA/IIB balance, a small-scale genetic screen for possible candidates identified Dunce (cAMP phosphodiesterase). In dunce mutants (or later with PKA overexpression), the IIA levels are increased and the IIB levels are reduced. Since PKA pathway has been suggested to downregulate quantal size in a previous study (Davis, 1998), they propose that the increase of postsynaptic IIA levels is compensatory to the reduced quantal size.

Overall, the study is very interesting, aiming to uncover a well-known phenomenon in the field. The high-resolution images of GluRIIA and IIB localization in postsynapses (Fig 2) is very impressive, which could provide new directions of study. This is also the most improved part of the manuscript compared to a previous version I had the chance to review. Overall, I think there is significant improvement in the manuscript, which is suitable for publication with modifications as follow (mainly on data analysis and writing):

Reviewer 3 Comments for the Author:
1. The increase or decrease of one subunit (IIA or IIB) lead to reverse changes in another subunit, while the co-subunit level (IID) is unchanged. I wonder the negative correlation of IIA and IIB are confined by the total amount of IID available to assemble GluRs or for localization. Have authors tested whether manipulating IID levels could affect IIA and IIB levels or allow an increase of one subunit without compromising the other's level? If not, could this be discussed if it is a possible explanation?

Reply: A very good suggestion! We tested the GluRIIA and GluRIIB levels when each of the essential subunits of GluRs, GluRIIC, GluRIID or GluRIIE was knocked down by RNAi, we found that both GluRIIA and GluRIIB decreased dramatically. However, GluRIIA and GluRIIB did not decrease to the same extent. Instead, the balance of GluRIIA/GluRIIB shifted towards more GluRIIA, suggesting that the negative correlation of GluRIIA and GluRIIB may still work when the essential subunit was limited (Fig. S8). As GluRIIA is mainly responsible for the postsynaptic responses (Petersen et al., 1997), the relative increase in GluRIIA when an essential subunit of GluRs was knocked down might be a functional compensation for the decrease of synaptic strength.

© 2020. Published by The Company of Biologists under the terms of the Creative Commons Attribution License (https://creativecommons.org/licenses/by/4.0/).

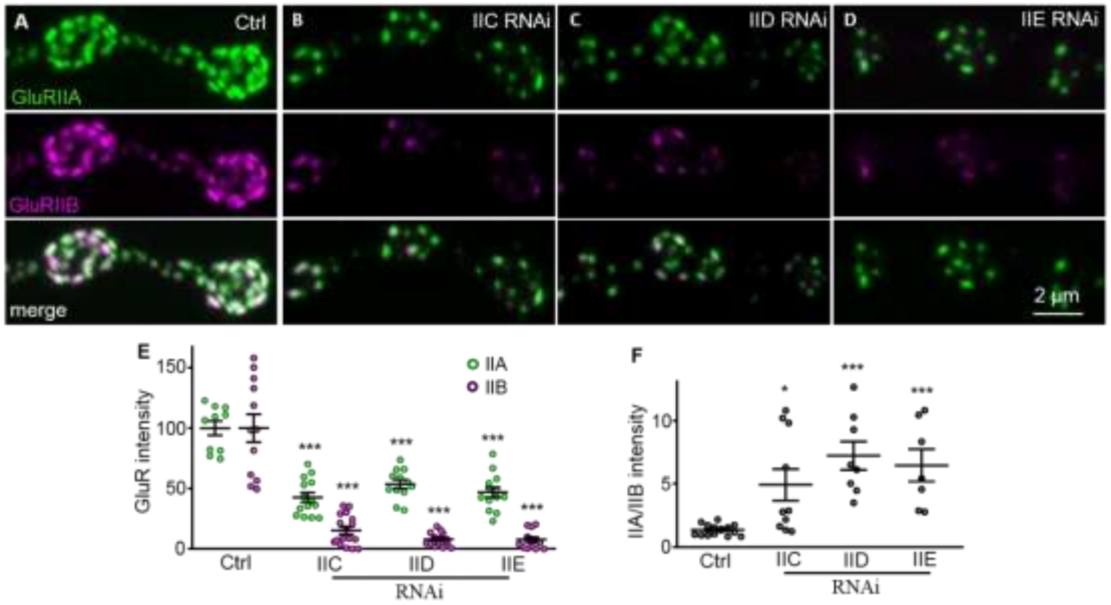

Fig. S8. Synaptic GluRIIA and GluRIIB decrease with the balance shifting towards more GluRIIA when an essential subunit of GluRs is knocked down. (A-D) Representative confocal images of late third-instar larval NMJ4 stained with anti-GluRIIA (green) and anti-GluRIIB (magenta). Full genotypes are as follows: Ctrl (*C57-Gal4/+*), IIC RNAi (*C57-Gal4/UAS-IIC RNAi*), IID RNAi (*C57-Gal4/UAS-IID RNAi*), and IIE RNAi (*C57-Gal4/UAS-IIE RNAi*). Scale bar: 2 µm. (E) Quantification of the intensities of anti-GluRIIA and anti-GluRIIB staining at the NMJ. Data are expressed as normalized staining intensities with respect to Ctrl (A). $n \geq 10$ for each genotype. (F) Quantification of the ratio of GluRIIA and GluRIIB intensities. *$p < 0.05$; ***$p < 0.001$. Error bars indicate the s.e.m.

2. It is very nice to see the better images of IIA and IIB forming concentric rings in postsynapses, with IIA taking the more central position. The morphology of the ring structures is depicted in Fig 2H, with connected four lobes for each ring. One question is that whether all IIA/IIB rings they observed are all in tetrameric forms? Are there other shapes or IIA only or IIB only rings (In particular when IIA/IIB ratios are altered)? Statistics is necessary to describe the percentages of clusters that are in tetrameric forms or in other forms. Also, the calculated lobe size (from Fig 2H) would be about 200 nm in diameter, comparing to the structure of a single glutamate receptor of less than 20 nm, it would be very informative to estimate the numbers of receptors in each lobe. When the lobe size changes with increases in IIA or IIB levels, the numbers of receptors would be changed accordingly. It would be nice if the authors could estimate the “numbers” of receptors in some plasticity model and describe that in Discussion, which would provide a more quantitative view.

Reply: As suggested, we have quantified the nanoclusters, i.e., lobes of subtype A and B receptor rings, for all genotypes and added in the results to the revised Figure 3 (Fig. 3K,L). The results showed that the nanocluster numbers of either subtype A or subtype B receptors were between 3 and 7. The number of the majority of type A and B receptor nanoclusters was 4 in wild type (Fig. 3K,L). The number of subtype A receptor nanoclusters was 5 when GluRIIA was overexpressed or GluRIIB was knocked down by RNAi (Fig. 3K). The number of subtype B nanoclusters was also 5 when GluRIIB was overexpressed or GluRIIA was knocked down by RNAi (Fig. 3L). These results indicate that the number of GluR nanoclusters was positively associated with the size of the rings. We have added these results in Results in lines 293 to 302.
Previous publications report that a PSD contains, on average, 60 GluRIIA and 60 GluRIIB complexes as determined by electrophysiological recordings (Schmid et al., 2008; Pawlu et al., 2004). Due to technical limitations, we were unable to measure the exact size of a single GluR tetramer. As the number of GluRIIA and GluRIIB nanoclusters changes in various manipulations, we predict that the number of GluR receptor molecules at each PSD will change accordingly.

3. It would be nice to show the Flag-tagged IIB images (immunostained by Flag antibody) in postsynapses, to reveal how the postsynaptic localization pattern compared to IIB immunostaining. If they are comparable, it would be a nice contribution to the field to use this Flag-IIB line.

© 2020. Published by The Company of Biologists under the terms of the Creative Commons Attribution License (https://creativecommons.org/licenses/by/4.0/).

Reply: We have performed co-staining of the NMJ terminals of homozygous GluRIIB- Flag lines with anti-GluRIIB together with anti-GluRIIA or anti-Flag. The staining patterns of Flag and IIB were perfectly matched. We have added this as Fig. S2 and described in the text "Co-immunostaining the NMJs of the homozygous *GluRIIB-Flag* lines with antibodies against GluRIIB and anti-GluRIIA or anti-Flag verified the synaptic localization of GluRIIB-Flag (Fig. S2)." in lines 317 to 319.

4. It appears that some part of this reciprocal regulation is at protein localization, not total protein levels, to the postsynapses. Some pathways are known to regulate IIA localization (or postsynaptic levels), which could be incorporated in the Discussion.
Reply: There are a few reports on the regulation of synaptic levels of specific GluR subunits. For example, subtype A receptors are anchored at the PSD by the actin- associated Coracle (Chen et al., 2005), and are regulated by a signaling pathway involving the Rho-type GEF (Pix) and its effector Pak kinase (Albin and Davis, 2004). Our recent studies also showed specific upregulation of GluRIIA but not GluRIIB when the calcium-dependent proteinase calpains are mutated (Metwally et al., 2019). We have included this point in Discussion in lines 578 to 584.

Reference

Albin, S.D.and Davis, G.W. (2004). Coordinating structural and functional synapse development: postsynaptic p21-activated kinase independently specifies glutamate receptor abundance and postsynaptic morphology. *J. Neurosci*. **24**, 6871-6879.
Chen, K. Y., Merino, C., Sigrist, S.J. and Featherstone, D. E. (2005). The 4.1 protein coracle mediates subunit-selective anchoring of *Drosophila* glutamate receptors to the postsynaptic actin cytoskeleton. *J. Neurosci*. **25**, 6667-6675.
Davis, G. W., DiAntonio, A., Petersen, S. A. and Goodman, C.S. (1998). Postsynaptic PKA controls quantal size and reveals a retrograde signal that regulates presynaptic transmitter release in Drosophila. Neuron 20, 305-315.
Lane, M. E. and Kalderon, D. (1993). Genetic investigation of cAMP-dependent protein kinase function in Drosophila development. Genes Dev 7, 1229-1243.
Marrus, S. B., Portman, S. L., Allen, M. J., Moffat, K. G. and DiAntonio, A. (2004). Differential localization of glutamate receptor subunits at the *Drosophila* neuromuscular junction. *J. Neurosci*. **24**, 1406-1415.
Menon, K. P., Andrews, S., Murthy, M., Gavis, E. R. and Zinn, K. (2009). The translational repressors Nanos and Pumilio have divergent effects on presynaptic terminal growth and postsynaptic glutamate receptor subunit composition. *J. Neurosci*. **29**, 5558-5572.
Menon, K.P., Sanyal, S., Habara, Y., Sanchez, R., Wharton, R. P., Ramaswami, M. and Zinn, K. (2004). The translational repressor Pumilio regulates presynaptic morphology and controls postsynaptic accumulation of translation factor eIF-4E. *Neuron* 44, 663-676.
Metwally, E., Zhao, G., Li, W., Wang, Q. and Zhang, Y.Q. (2019). Calcium-Activated Calpain Specifically Cleaves Glutamate Receptor IIA But Not IIB at the Drosophila Neuromuscular Junction. *J. Neurosci*. **39**, 2776-2791.
Pan, L. and Broadie, K. S. (2007). *Drosophila* fragile X mental retardation protein and metabotropic glutamate receptor A convergently regulate the synaptic ratio of ionotropic glutamate receptor subclasses. *J. Neurosci*. **27**, 12378-12389.
Pawlu, C., DiAntonio, A. and Heckmann, M. (2004). Postfusional control of quantal current shape. *Neuron* **42**, 607-618.
Petersen, S.A., Fetter, R.D., Noordermeer, J.N., Goodman, C.S., and DiAntonio, A. (1997). Genetic analysis of glutamate receptors in Drosophila reveals a retrograde signal regulating presynaptic transmitter release. Neuron 19, 1237–1248.
Schmid, A., Hallermann, S., Kittel, R. J., Khorramshahi, O., Frolich, A. M., Quentin, C., Rasse, T. M., Mertel, S., Heckmann, M. and Sigrist, S. J. (2008). Activity-dependent site specific changes of glutamate receptor composition in vivo. *Nat. Neurosci*. **11**, 659-666.
Sigrist, S. J., Thiel, P. R., Reiff, D. F., Lachance, P. E., Lasko, P. and Schuster, C. M. (2000). Postsynaptic translation affects the efficacy and morphology of neuromuscular junctions. *Nature* 405, 1062-1065.
Sigrist, S. J., Reiff, D. F., Thiel, P. R., Steinert, J. R. and Schuster, C. M. (2003). Experience-dependent strengthening of Drosophila neuromuscular junctions. J. Neurosci. 23, 6546-6556.
Sulkowski, M., Kim, Y. J. and Serpe, M. (2014). Postsynaptic glutamate receptors regulate local BMP signaling at the *Drosophila* neuromuscular junction. *Development* **141**, 436-447.

© 2020. Published by The Company of Biologists under the terms of the Creative Commons Attribution License (https://creativecommons.org/licenses/by/4.0/).

Zhong, Y., Budnik, V. and Wu, C. F. (1992). Synaptic plasticity in *Drosophila* memory and hyperexcitable mutants: role of cAMP cascade. *J. Neurosci*. **12**, 644- 651.

Second decision letter

MS ID#: DEVELOP/2020/191874

MS TITLE: Postsynaptic cAMP signalling regulates the antagonistic balance of glutamate receptor subtypes

AUTHORS: Kai Zhao, Huilin Hong, Lu Zhao, Sheng Huang, Ying Gao, Elsayed Metwally, Yuqiang Jiang, Stephan J Sigrist, and Yong Q Zhang
ARTICLE TYPE: Research Article

I am delighted to tell you that your manuscript has been accepted for publication in Development, pending our standard ethics checks.

Reviewer 1

Advance summary and potential significance to field

Regulation of Glutamate receptor subtype competition at the Drosopphila NMJ.

Comments for the author

The authors addressed most of my concerns in their revision. Per addressing the question of active vs passive negative regulation of IIA/IIB, they performed a knockdown of an obligatory GluR subunit and demonstrated that the IIA/IIB ratio is altered to preferentially retain an appropriate amount of IIA. Their logic is that if passive competition for binding was the only factor at play, the ratio of IIA:IIB would have been maintained in this situation. I agree with this general logic.

Per my concern that PKA was not mediating the balance but rather mediating IIA on its own, the authors performed the suggested experiment - they knocked down PKA in the IIA and IIB null animals. Based on these results, they show that postsynaptic PKA is not required for the antagonistic balance. This seems in contrast with their summary statement that the antagonistic balance of GluR subtypes is regulated by postsynaptic cAMP signaling, so they might want to clean that up.

It's also nice to see that IIB-Flag signal on the western blot is significantly reduced upon GluRIIB-RNAi expression. This demonstrates that reductions in the IIB level are within the range of detection of this method.

Overall, I'm satisfied with the authors' revisions.

© 2020. Published by The Company of Biologists under the terms of the Creative Commons Attribution License (https://creativecommons.org/licenses/by/4.0/).
